# Quantitative morphological phenotyping of infection structures in cucumber downy mildew and powdery mildew

**DOI:** 10.3389/fpls.2026.1844862

**Published:** 2026-07-13

**Authors:** Yi Wu, Zonghuan Han

**Affiliations:** 1 School of Computing and Data Science, Xiamen University Malaysia, Sepang, Selangor, Malaysia; 2 College of Information and Electrical Engineering, China Agricultural University, Beijing, China

**Keywords:** cucumber pathogens, disease diagnosis, infection structures, instance segmentation, morphological phenotyping, precision disease management

## Abstract

**Introduction:**

Cucumber diseases severely affect yield and quality. Deep learning-based analysis of microscopic pathogen images enables high-throughput identification and counting of pathogens, thereby facilitating early disease detection. However, most existing pathogen-recognition methods focus mainly on qualitative identification and cannot quantitatively characterize pathogen morphology, which limits their ability to reveal the developmental characteristics and functional differentiation of different infection structures from the perspective of pathogen morphology–function adaptability.

**Methods:**

To address this issue, this study focused on cucumber powdery mildew and downy mildew and achieved precise extraction and characterization of pathogen morphological features based on microscopic image instance segmentation. First, an in situ stained microscopic image dataset of cucumber pathogens was constructed. Second, an instance segmentation model, SWS-YOLO11n, was developed for cucumber pathogen infection structures to accurately identify and segment different infection structures in microscopic images. Finally, morphological analysis methods were used to quantitatively extract and characterize pathogen infection-structure features.

**Results:**

Experimental results showed that SWS-YOLO11n achieved a detection mAP@0.5 of 97.4% and a segmentation mAP@0.5 of 96.2%, with a model size of only 5.6 MB. The extraction accuracy of perimeter, area, and curvature reached R^2^ values greater than 0.90. In addition, category-wise morphological distribution analysis showed that different infection-structure types exhibited clear differentiation in size, contour complexity, and elongation.

**Discussion:**

This study provides an effective tool for high-throughput phenotyping of cucumber pathogen infection structures. The proposed method offers methodological support for disease diagnosis, pathogen morphological phenotyping, and precision disease management in horticultural production.

## 1 Introduction

Cucumber is an important cucurbit crop, and its yield and quality are closely linked to agricultural economic stability and farmers’ income (Liao et al., 2026; Han et al., 2026). Powdery mildew and downy mildew are two major diseases affecting cucumber production, caused by *Podosphaera xanthii* and *Pseudoperonospora cubensis*, respectively (Alomran et al., 2023). Both pathogens are primarily dispersed via air currents, have short incubation periods, and can initiate multiple infection cycles. Missing the early control window often leads to rapid spread and large-scale outbreaks, posing a significant threat to the stable development of the cucumber industry (Ishii et al., 2001). Microscopic imaging provides high sensitivity for early pathogen detection and enables fine-grained morphological assessment that is not achievable through macroscopic or field-level phenotyping. Consequently, quantitative characterization of infection structures based on microscopic imagery is a practical and necessary approach for understanding pathogen development and improving early warning and management strategies.

Many studies have investigated cucumber disease diagnosis and pathogen characterization. Early computer-vision-based approaches primarily relied on traditional image-processing techniques. Sasaki et al. (1999) identified cucumber anthracnose by combining leaf reflectance characteristics with optical filtering, demonstrating feasibility but limited accuracy. Wu and Wang (Wu and Wang, 2017) extracted color-feature parameters at different disease stages to distinguish anthracnose from brown spot disease effectively. With advances in deep learning, more powerful diagnostic frameworks have emerged. Liu et al. (Liu et al., 2025) proposed an intelligent deep learning architecture for precision vegetable disease detection, demonstrating the potential of advanced deep learning methods to improve the accuracy and intelligence of disease diagnosis in modern agriculture. Wang et al. (Wang et al., 2014) developed the DUNet model by integrating DeepLabV3+ and U-Net, and achieved a disease-severity classification accuracy of 92.85% under complex background conditions. Zhao et al. (Zhao et al., 2022) developed the DTL-SE-ResNet50 model based on transfer learning and attention mechanisms. This method required only 0.13 s to process a single image and improved the recognition accuracy to 97.24%, thereby significantly enhancing diagnostic efficiency and reliability.

In deep-learning-based microscopic image analysis, researchers have developed model optimizations tailored to microbial targets. Li et al. (Li et al., 2023) proposed the MG-YOLO algorithm, which integrates multi-head self-attention and BiFPN feature fusion, achieving a spore-detection accuracy of 0.983 and an inference time of 0.009s per image for Botrytis cinerea spores. Qiao et al. (Qiao et al., 2025) enhanced YOLOv5 by incorporating the NAM attention mechanism and a weight-sparsity penalty strategy, resulting in an mAP@0.5 of 95.80% for the detection of *Pseudoperonospora cubensis* spores. For microscopic image segmentation, several studies have addressed challenges such as uneven illumination and overlapping microbial structures. Zhang et al. (Zhang et al., 2024) introduced the CRF_ResUNet++ model based on UNet++, combining ResNet with fully connected conditional random fields to segment Fusarium spores, achieving an F1-score of 0.943. Zhao et al. (Zhao et al., 2019) proposed a Constrained Focal Loss (CFL) that strengthens attention to spore-contour pixels, enabling the DeepLabv3+ model to reach an average intersection over union (IoU) of 91.0% and providing a technical reference for pathogen segmentation in microscopic imagery.

In computer-vision-based pathogen morphology analysis, researchers have focused on developing new feature types and extraction strategies. Bangun et al. (Bangun et al., 2016) quantified morphological descriptors such as area, perimeter, and compactness, demonstrating that compactness and circularity can effectively differentiate among pathogen species. Seo et al. (Seo et al., 2018) calculated nine morphological attributes, including major-axis length and eccentricity, from hyperspectral images to classify fifteen foodborne bacterial species. Javidan et al. (Javidan et al., 2024) combined texture, color, and shape features with the Butterfly Optimization Algorithm (BOA), achieving a recognition accuracy of 95% for fungal spores causing tomato diseases. Oh et al. (Oh et al., 1996) developed an automated imaging system that decomposes spore contours based on curvature metrics, enabling quantitative assessment of fungal spore germination characteristics and providing a useful tool for analyzing pathogen biological traits.

Despite notable progress in previous studies, the extraction of infection-structure features from microscopic images of cucumber pathogens remains insufficiently explored. First, most existing algorithms focus on a single pathogen or a single infection stage, limiting their applicability in field conditions where mixed infections involving multiple pathogens are common. Consequently, the coverage of pathogen diversity and infection-structure variability remains inadequate. Second, feature analysis is often restricted to spore counting, without integrating plant pathology knowledge to construct biologically meaningful quantitative frameworks, leaving the deeper analytical value of microscopic imagery underutilized. To address these challenges, this study focuses on powdery mildew and downy mildew of cucumber and develops an instance-segmentation–based approach for precise extraction of pathogen morphological features from microscopic images. The main contributions are as follows:

1. A microscopic image dataset of in situ–stained cucumber pathogens was constructed, systematically documenting the morphological characteristics of various infection structures of downy mildew and powdery mildew across their developmental stages.
2. A microscopic image instance-segmentation model, SWS-YOLO11n, was developed. Using YOLO11n as the baseline, SA attention was incorporated to enhance small-object detection, WTConv was integrated to improve both fine-detail and global feature extraction efficiency, and SHSA attention was employed to reduce computational redundancy.
3. A precise morphological-feature extraction method for cucumber pathogens was established. Based on the high-accuracy masks generated by SWS-YOLO11n, and combined with morphological image-analysis techniques, the approach achieves accurate extraction of area, perimeter, curvature, and other key morphological attributes of diverse infection structures.
4. A category-wise statistical analysis of the morphological distributions of six infection-structure types was conducted, revealing their differentiation patterns in area, perimeter, curvature, and ellipticity, and providing quantitative support for understanding the developmental characteristics and functional differentiation of cucumber pathogen infection structures from the perspective of “morphology–function” adaptability.

## 2 Materials and methods

### 2.1 An infection-cycle-based strategy for extracting morphological features of cucumber pathogens

**Figure 1:**
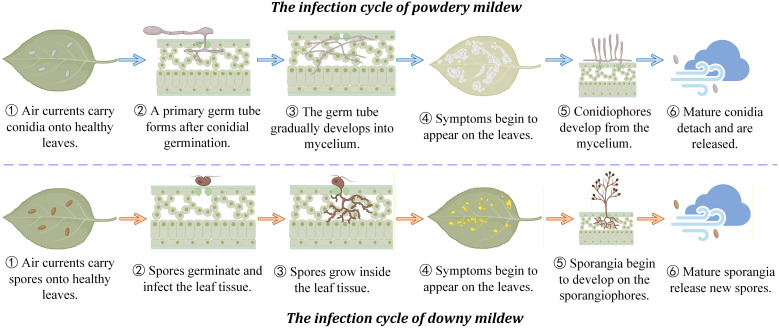
Infection cycles of cucumber powdery mildew and downy mildew.

Cucumber powdery mildew and downy mildew are two common diseases that follow distinct but biologically comparable infection cycles, as illustrated in **Figure 1**. It should be noted that *Podosphaera xanthii*, a major causal agent of cucumber powdery mildew, belongs to the true fungi, whereas *Pseudoperonospora cubensis*, the causal agent of cucumber downy mildew, is an oomycete (Zhang et al., 2022; Bahmani et al., 2025). Accordingly, the two pathogens differ in several biological characteristics. Powdery mildew pathogens mainly produce conidia, lack a motile zoospore stage, and usually grow on the leaf surface and penetrate epidermal cells directly to form haustoria. In contrast, downy mildew pathogens mainly produce sporangia and may release motile zoospores under high humidity, typically infecting host tissues through stomata. Despite these differences, both pathogens form distinct microscopic infection structures and are therefore suitable targets for the present analysis. Both pathogens exhibit typical characteristics of obligate parasitism throughout their infection processes. Airborne propagules adhere to the leaf surface, absorb moisture, germinate to produce germ tubes, and form appressoria. Infection pegs are then generated to penetrate the cuticle and epidermis through enzymatic degradation and mechanical pressure. Haustoria are formed to extract nutrients from host cells, while primary hyphae extend and branch within the tissue. In powdery mildew, penetration typically occurs directly through the epidermis. Hyphae spread across the leaf surface and differentiate upright conidiophores, which continuously generate and release conidia, enabling repeated secondary infections. In downy mildew, sporangia release zoospores under high-humidity conditions. Zoospores migrate along the water film to stomatal regions, lose their flagella, germinate, and invade through appressoria and infection pegs. Hyphae predominantly grow intercellularly and produce haustoria. Under favorable conditions, sporangiophores emerge through stomata, form terminal sporangia, and disseminate through wind or rain splash, enabling subsequent cycles of zoospore release. Both pathogens complete multiple infection cycles by continuously producing reproductive structures and propagating across host tissues.

**Figure 2:**
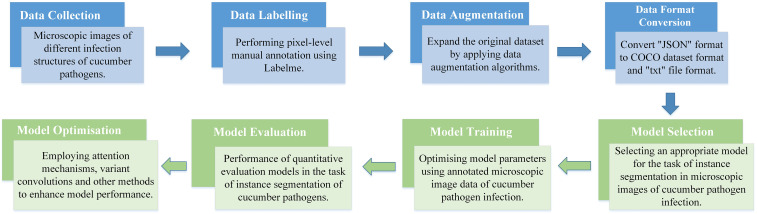
Flowchart of deep learning-based segmentation of microscopic images of cucumber pathogen infection.

The deep-learning workflow for instance segmentation of cucumber pathogen infection structures in microscopic images consists of data acquisition, data annotation, data augmentation, data-format conversion, model selection, model training, model evaluation, and model optimization, as illustrated in **Figure 2**.

### 2.2 Data acquisition and pre-processing

#### 2.2.1 Data acquisition

**Figure 3:**
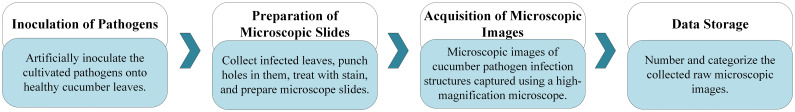
Flowchart for data acquisition of microscopic images of cucumber pathogen infection.

The dataset constructed in this study covers microscopic images of cucumber pathogen infection at different developmental stages. The data acquisition workflow includes pathogen inoculation, preparation of microscopic slides, microscopic image collection, and data storage, as illustrated in **Figure 3**.

##### 2.2.1.1 Pathogen inoculation

Pathogens were artificially inoculated onto healthy cucumber leaves to allow colonization and infection, simulating the natural disease cycle. Disease progression was monitored throughout the incubation period. After inoculation, leaves were maintained under suitable environmental conditions, routinely observed, and recorded at different infection stages.

##### 2.2.1.2 Microscope slide preparation

Once infection was established, representative symptomatic regions were selected to prepare microscope slides for subsequent observation and morphological analysis. Slide preparation followed several steps. First, samples were collected from lesion areas using a sterilized punch to ensure that intact infection structures were included. Second, the sampled leaf tissues were immersed in a fixative to preserve their original morphology and prevent deformation of infection structures. Third, a clearing solution was applied to remove native pigments and reduce background interference, improving subsequent staining quality. Fourth, staining was performed to enhance contrast and highlight pathogen infection structures for microscopic imaging. Finally, a coverslip was applied and sealed with a mounting medium to prevent drying and contamination, ensuring long-term preservation and imaging stability.

##### 2.2.1.3 Microscopic image acquisition

Images of pathogen infection structures were captured using an Olympus BX51 microscope. Appropriate magnification was selected based on the size of pathogen structures. In this study, imaging was performed at 400× magnification (40× objective, 10× eyepiece) to obtain clear microscopic details. The digital camera connected to the microscope was a DigiRetina 16, featuring a 1/2.33-inch sensor, 16 megapixels effective resolution, and a pixel size of 1.335 μm × 1.335 μm. The resulting images had a resolution of 1280 × 960 pixels and were saved in JPG format.

##### 2.2.1.4 Data storage

**Figure 4:**
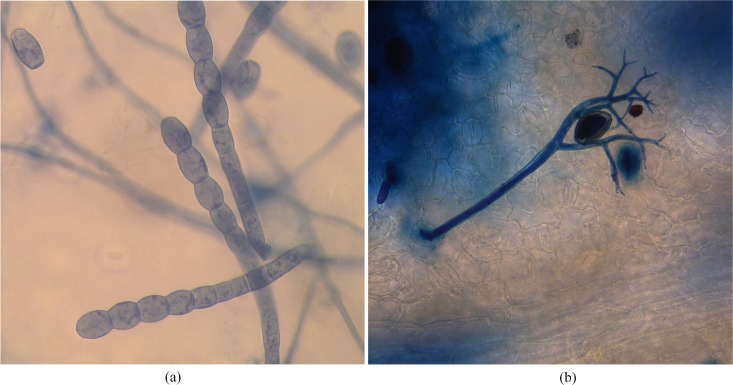
Microscopic images of pathogen infection structures. **(a)** Microscopic images of infection structures of powdery mildew pathogens. **(b)** Microscopic images of infection structures of downy mildew pathogens.

To ensure long-term usability and standardized management, all images were systematically archived and categorized according to pathogen species and cultivation time for subsequent analysis and application (**Figure 4**).

#### 2.2.2 Data annotation and augmentation

A total of 1,197 original microscopic images were annotated at the pixel level, including 564 images of cucumber powdery mildew and 633 images of cucumber downy mildew. Six categories of infection structures were labeled for instance segmentation, including PM-Spore, PM-Conidium, PM-Conidiophore, DM-Sporangium, DM-Sporangiophore, and DM-Hyphae. Here, PM and DM denote powdery mildew and downy mildew, respectively. In total, 10,303 infection-structure instances were annotated, and the number of instances in each category is summarized in **Table 1**.

**Table 1:** Annotation counts of different infection-structure categories in the cucumber pathogen dataset.

Label	Amount
PM-Spore	1536
PM-Conidium	4314
PM-Conidiophore	1796
DM-Sporangium	733
DM-Sporangiophore	667
DM-Hyphae	1257

To ensure annotation quality and consistency, all images were annotated using Labelme 5.5.0 by two uniformly trained annotators under the guidance of two plant pathologists from the Institute of Plant Protection, Tianjin Academy of Agricultural Sciences. Before annotation, a unified annotation protocol was established, including the definition of each infection-structure category, boundary delineation criteria, and rules for handling overlapping or ambiguous regions. After the initial annotation, all labels were reviewed image by image by the two plant pathology experts. Labels with unclear boundaries, inconsistent category assignment, or insufficient annotation quality were revised or discarded as necessary to minimize subjective bias.

To further evaluate annotation reliability, 24 images were randomly selected from the dataset and independently re-annotated by the two annotators. The mean inter-annotator IoU across the six infection-structure categories was 0.8727, indicating good annotation consistency. Relatively lower agreement was mainly observed in regions with overlapping hyphae, densely branched sporangiophores, and weakly defined object boundaries. For these ambiguous samples, the final annotations were determined through joint discussion between the annotators and the plant pathology experts.

After annotation, the dataset was divided into training, validation, and test sets at a ratio of 8:1:1, resulting in 958 images for training, 120 images for validation, and 119 images for testing.

**Figure 5:**
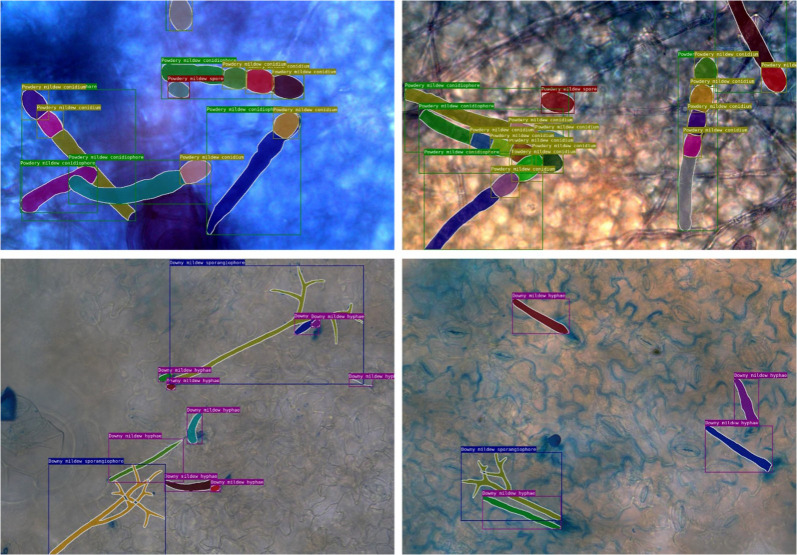
Visualization of the data annotation results.

The visualization of the annotation results is shown in **Figure 5**.

### 2.3 SWS-YOLO11n-based instance segmentation model for cucumber pathogen infection structures

#### 2.3.1 Overall architecture of the SWS-YOLO11n instance segmentation model

**Figure 6:**
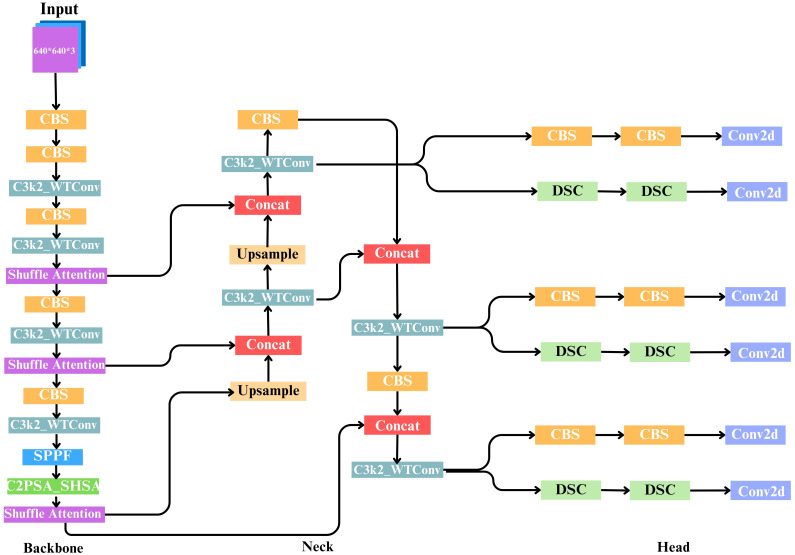
Schematic diagram of the SWS-YOLO11n architecture.

The YOLO model family has been widely applied in computer vision due to its efficient object detection and instance segmentation capabilities (Jiang et al., 2022). However, the original YOLO11 model still faces challenges when dealing with complex microscopic imagery, including limited feature-extraction capacity and reduced accuracy in recognizing small targets. These limitations hinder the precise segmentation of pathogen infection structures. To address these issues, YOLO11 was enhanced according to the characteristics of microscopic images of cucumber pathogens, aiming to improve segmentation accuracy and model robustness. The architecture of the proposed SWS-YOLO11n network is shown in **Figure 6**.

#### 2.3.2 SA attention mechanism

**Figure 7:**
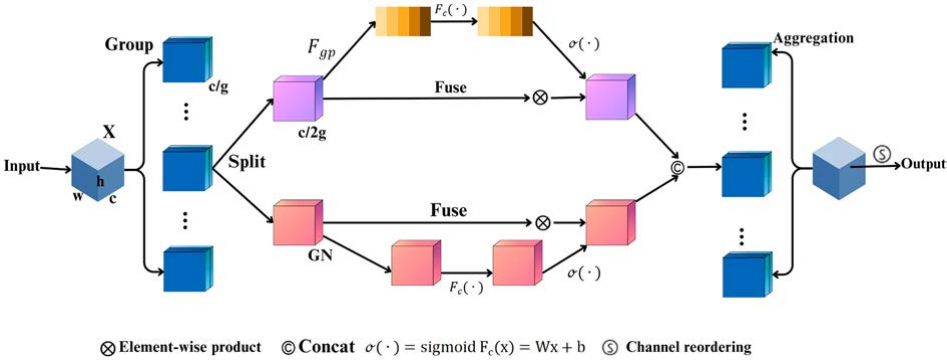
Shuffle attention module structure diagram.

Shuffle Attention (SA) is a lightweight and efficient attention mechanism based on channel rearrangement, designed for deep convolutional neural networks (Zhang and Yang, 2021). Microscopic images of cucumber pathogens typically contain fine-grained infection structures with complex morphology and subtle variations. SA facilitates effective information interaction across channels, enabling the model to better capture and represent these subtle pathogen features. This channel interaction not only strengthens attention to fine details but also reduces information loss between channels, thereby improving the detection performance for small-scale targets such as conidia and sporangia. The SA attention mechanism consists of four main components: feature grouping, channel attention, spatial attention, and feature aggregation. Its basic architecture is illustrated in **Figure 7**.

The main concept of SA is to split the input feature map along the channel dimension into multiple sub-features, and then perform channel attention and spatial attention on each sub-feature separately. For an input feature map $X\in {\mathbb{R}}^{C\times H\times W}$, where C, H, and W denote the channel number, spatial height, and spatial width, respectively, SA first divides X into Ggroups along the channel dimension, which can be written as $X=[X_{1},\cdots \;,X_{G}]$. Each sub-feature $X_{k}\in {\mathbb{R}}^{\frac{C}{G}\times H\times W}$gradually learns task-related semantic information during training. Within each attention unit, $X_{k}$ is further split into two branches along the channel dimension, denoted as $X_{k1},X_{k2}\in {\mathbb{R}}^{\frac{C}{2G}\times H\times W}$. One branch is used to capture inter-channel dependencies and generate a channel attention map, whereas the other is responsible for modeling spatial dependencies and producing a spatial attention map. Through this two-branch structure, the model can simultaneously focus on both the most informative feature content and the most relevant spatial locations.

In the channel-attention branch, Global Average Pooling (GAP) (Hsiao et al., 2019) is applied to generate the channel-wise statistical descriptor $\text{s}\in {\mathbb{R}}^{\text{C}/2\text{G}\times 1\times 1}$. This descriptor is obtained by reducing ${\text{X}}_{\text{k}1}\;$along the spatial dimensions H×W. The computation is defined in Equation 1:

(1) $$\text{s}={\text{F}}_{\text{gp}}({\text{X}}_{\text{k}1})=\frac{1}{\text{H}\times \text{W}}\sum_{\text{i}=1}^{\text{H}}\sum_{\text{j}=1}^{\text{W}}{\text{X}}_{\text{k}1}(\text{i},\text{j})$$

A simple gating mechanism activated by the sigmoid function is then applied to adaptively select the most informative channels. The final output of the channel-attention branch is computed as shown in Equation 2:

(2) $${\text{X}}_{\text{k}1}^{'}=\text{σ}({\text{F}}_{\text{c}}(\text{s}))\cdot {\text{X}}_{\text{k}1}=\text{σ}({\text{W}}_{1}\text{s}+{\text{b}}_{1})\cdot {\text{X}}_{\text{k}1}$$

where ${\text{W}}_{1}\in {\mathbb{R}}^{\text{C}/2\text{G}\times 1\times 1}$ and ${\text{b}}_{1}\in {\mathbb{R}}^{\text{C}/2\text{G}\times 1\times 1}$ are the parameters used to scale and shift s.

Unlike channel attention, spatial attention focuses on identifying the location of important regions within the feature map, serving as a strong complement to the channel-attention branch. During spatial-attention processing, Group Normalization (GN) is first applied to ${\text{X}}_{\text{k}2}$ to generate spatial statistical information. The representation of ${\text{ X}}_{\text{k}2}$ is then enhanced through the operator ${\text{ F}}_{\text{c}}(\cdot )$, producing compact features analogous to those generated in the channel-attention branch. The final output of the spatial-attention branch is given by Equation 3:

(3) $${\text{X}}_{\text{k}2}^{'}=\text{σ}({\text{W}}_{2}\cdot \text{GN}({\text{X}}_{\text{k}2})+{\text{b}}_{2})\cdot {\text{X}}_{\text{k}2}$$

where ${\text{W}}_{2}$ and ${\text{b}}_{2}$ are learnable parameters of size ${\mathbb{R}}^{\text{C}/2\text{G}\times 1\times 1}$. The outputs of the two branches are then concatenated along the channel dimension to restore the original channel size, as shown in Equation 4:

(4) Xk'=[Xk1',Xk2']∈ℝC/G×H×W

#### 2.3.3 WTConv

Wavelet Convolution (WTConv) is a method that integrates the Wavelet Transform (WT) with convolutional neural networks to enlarge the receptive field through multi-scale analysis while maintaining high parameter efficiency (Fujieda et al., 2018; Finder et al., 2024).

In microscopic images of cucumber pathogens, structural features vary across different spatial scales and frequency components. Traditional convolution relies on kernels of fixed size, which limits its ability to capture features at multiple scales. By applying the wavelet transform, WTConv extracts image representations at different scales, allowing effective multi-scale feature capture. Moreover, the infection structures of cucumber pathogens exhibit distinct characteristics across frequency bands. The wavelet transform decomposes an image into multiple frequency components, enabling separate processing of low-frequency information (e.g., large-scale pathogen structures) and high-frequency information (e.g., fine details, edges, and textures). This facilitates the simultaneous acquisition of both global structure and local detail, enabling WTConv to recognize more complex pathogen features. Because WTConv can directly replace deep convolutional layers in CNNs, it is well-suited for integration with the C3k2 module, further enhancing the network’s feature-extraction capability.

The core idea of WTConv is to integrate the time–frequency analysis capability of the wavelet transform with convolution operations to overcome the limitation of the local receptive field in traditional convolutional neural networks. This integration enables CNNs to better capture global contextual information and improves overall feature-extraction performance. The WT is a mathematical technique used to transform a signal from the time domain into the frequency domain. Unlike the Fourier transform, WT provides joint localization in both time and frequency, thus supporting multi-scale analysis (Pal et al., 2022). WTConv employs the Haar wavelet transform for computation. For a given image X, a single-level Haar WT decomposition along one spatial dimension is implemented using depthwise convolutions with kernels $[1,1]/\sqrt{2}$ and $[1,-1]/\sqrt{2}$ followed by standard downsampling with a factor of 2. To perform a two-dimensional Haar WT, the operation must be applied along both spatial dimensions. Consequently, four depthwise convolution kernels with a stride of 2 are used, as defined in Equation 5, and the corresponding kernel sets are shown in Equation 6.

(5) $${\text{f}}_{\text{LL}}=\frac{1}{2}[\begin{array}{cc} 1 & 1\\ 1 & 1 \end{array}],{\text{f}}_{\text{LH}}=\frac{1}{2}[\begin{array}{cc} 1 & -1\\ 1 & -1 \end{array}],{\text{f}}_{\text{HL}}=\frac{1}{2}[\begin{array}{cc} 1 & 1\\ -1 & -1 \end{array}],{\text{f}}_{\text{HH}}=\frac{1}{2}[\begin{array}{cc} 1 & -1\\ -1 & 1 \end{array}]$$

here, ${\text{f}}_{\text{LL}}\;$is a low-pass filter, whereas ${\text{f}}_{\text{LH}}$, ${\text{f}}_{\text{HL}}$ and ${\text{f}}_{\text{HH}}$ are high-pass filters. For each input channel, the depthwise convolution produces four output channels, each retaining the same spatial resolution as X. The computation is defined in Equation 6:

(6) $$\left[{\text{X}}_{\text{LL}},{\text{X}}_{\text{LH}},{\text{X}}_{\text{HL}},{\text{X}}_{\text{HH}}]=\text{Conv}([{\text{f}}_{\text{LL}},{\text{f}}_{\text{LH}},{\text{f}}_{\text{HL}},{\text{f}}_{\text{HH}}],\text{X}\right)$$

here, ${\text{X}}_{\text{LL}}\;$represents the low-frequency component of X, while ${\text{X}}_{\text{LH}}$, ${\text{X}}_{\text{HL}}$ and ${\text{ X}}_{\text{HH}}$ correspond to the horizontal, vertical, and diagonal high-frequency components, respectively, yielding four frequency-band feature maps. Since the convolution kernels used in Equation 5 form a standard orthogonal basis, the Inverse Wavelet Transform (IWT) can be obtained through transposed convolutions. The reconstruction is shown in Equation 7:

(7) $$\text{X}=\text{Conv}-\text{transposed}([{\text{f}}_{\text{LL}},{\text{f}}_{\text{LH}},{\text{f}}_{\text{HL}},{\text{f}}_{\text{HH}}],[{\text{X}}_{\text{LL}},{\text{X}}_{\text{LH}},{\text{X}}_{\text{HL}},{\text{X}}_{\text{HH}}])$$

**Figure 8:**
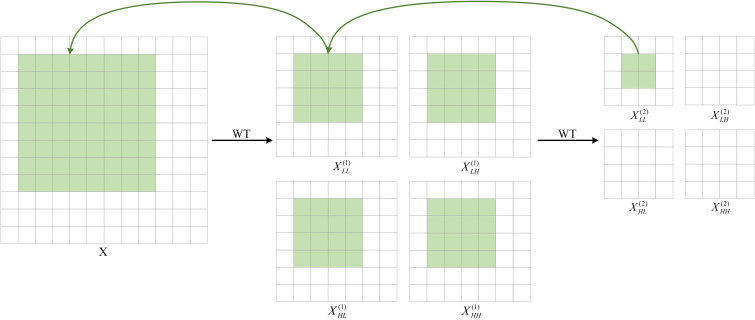
Wavelet transform in convolution.

A cascaded (multi-level) wavelet decomposition can then be obtained by recursively decomposing the low-frequency component. The decomposition at each level is defined in Equation 8:

(8) $${\text{X}}_{\text{LL}}^{\left(\text{i}\right)},{\text{X}}_{\text{LH}}^{\left(\text{i}\right)},{\text{X}}_{\text{HL}}^{\left(\text{i}\right)},{\text{X}}_{\text{HH}}^{\left(\text{i}\right)}=\text{WT}({\text{X}}_{\text{LL}}^{\left(\text{i}-1\right)})$$

here, ${\text{X}}_{\text{LL}}^{\left(0\right)}=\text{X}$ and i denotes the current decomposition level. As the level increases, the frequency resolution of the low-frequency component improves, whereas its spatial resolution decreases. Performing convolution in the wavelet domain, therefore, yields a much larger effective receptive field. As illustrated in **Figure 8**, applying a 2×2 convolution kernel to the second-level low-frequency sub-band ${\text{X}}_{\text{LL}}^{\left(2\right)}$ requires only four parameters, yet the kernel responds to low-frequency information within an 8×8 receptive field in the original input X.

Because enlarging the convolution kernel causes the number of parameters to increase quadratically, the input feature map is first decomposed by a wavelet transform into low-frequency and high-frequency components while performing downsampling. Small-kernel depthwise separable convolutions are subsequently conducted on these frequency components separately, after which the final feature representation is recovered through the inverse wavelet transform. The overall procedure can be formulated in Equation 9:

(9) Y=IWT(Conv(W,WT(X)))

here, X denotes the input tensor, and W represents a k×k depthwise convolution kernel whose input channel dimension is four times that of X. This design not only separates the convolution operations among different frequency components, but also allows a small convolution kernel to cover a considerably larger effective area in the original input space, thereby significantly enlarging the receptive field with respect to X. Following the cascading mechanism described in Equation 8, this single-level hybrid operation can be further generalized in a recursive manner. The recursive form is given in Equation 10 and Equation 11:

(10) $${\text{X}}_{\text{LL}}^{\left(\text{i}\right)},{\text{ X}}_{\text{H}}^{\left(\text{i}\right)}=\text{WT}({\text{X}}_{\text{LL}}^{\left(\text{i}-1\right)})$$

(11) $${\text{Y}}_{\text{LL}}^{\left(\text{i}\right)},{\text{ Y}}_{\text{H}}^{\left(\text{i}\right)}=\text{Conv}({\text{W}}^{\left(\text{i}\right)},({\text{X}}_{\text{LL}}^{\left(\text{i}\right)},{\text{X}}_{\text{H}}^{\left(\text{i}\right)}))$$

here, $X_{LL}^{\left(0\right)}$represents the input feature map of the current layer, while $X_{H}^{\left(i\right)}$denotes the three high-frequency components at the i-th decomposition level. To integrate the outputs from multiple frequency levels, the linear properties of the WT and inverse wavelet transform can be exploited, as shown in Equation 12:

(12) IWT(X+Y)=IWT(X)+IWT(Y)

Therefore, the final result of wavelet-domain convolution is obtained by summing the convolution outputs across all decomposition levels. The overall computation is shown in Equation 13:

(13) $${\text{Z}}^{\left(\text{i}\right)}=\text{IWT}({\text{Y}}_{\text{LL}}^{\left(\text{i}\right)}+{\text{Z}}^{\left(\text{i}+1\right)},{\text{Y}}_{\text{H}}^{\left(\text{i}\right)})$$

here, ${\text{Z}}^{\left(\text{i}\right)}\;$denotes the aggregated output after level i.

#### 2.3.4 SHSA attention mechanism

**Figure 9:**
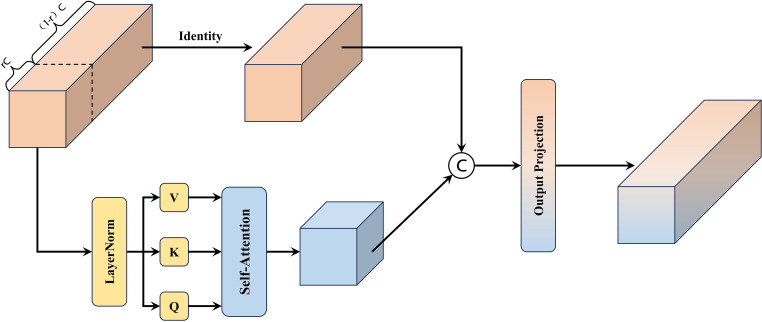
Diagram of the single-head self-attention structure.

Single-Head Self-Attention (SHSA) is a variant of the self-attention mechanism (Zhang et al., 2023; Shaw et al., 2018). Self-attention models the relationships among input elements to capture global contextual information and long-range dependencies, and it forms a core component of Transformer architectures (Qian et al., 2022). The basic structure is shown in **Figure 9**. Unlike traditional multi-head self-attention, SHSA employs only a single attention head to aggregate spatial features rather than multiple heads. This design eliminates the computational redundancy inherent in multi-head attention and reduces memory access overhead. In the instance segmentation of cucumber pathogen microscopic images, the SHSA mechanism enhances model performance by modeling interactions among different spatial regions of the image. It is particularly effective in fine-grained feature analysis and in capturing long-range dependencies.

Multi-Head Attention leverages multiple attention heads to learn diverse representations and dependency patterns, thereby enabling the capture of richer feature information (Voita et al., 2019). However, experimental observations have shown that, particularly in the later stages of deep networks, multi-head attention often exhibits redundancy. During feature extraction and prediction, this redundancy leads to reduced computational efficiency and increased memory access cost. Further studies indicate that removing some attention heads does not significantly degrade model accuracy; in some cases, eliminating a single attention head may even slightly improve segmentation performance. Therefore, the SHSA design adopts a single-head attention mechanism to fundamentally avoid the redundancy introduced by multi-head structures, ensuring a more streamlined and efficient training and inference process.

The core idea of SHSA is that each feature computes its relevance to other features by comparing its Query ( Q) with their Keys ( K), and then aggregates the corresponding Values ( V) using these relevance weights to obtain a new representation. “Single-Head” indicates that only one set of attention weights is used, rather than multiple parallel sets as in Multi-Head Attention. The computation of SHSA is defined as follows:

First, the input feature map X is divided along the channel dimension into two parts, ${\text{X}}_{\text{att}}$ and ${\text{X}}_{\text{res}}$. The operation is defined in Equation 14:

(14) $${\text{X}}_{\text{att}},\;{\text{X}}_{\text{res}}=\text{Split}(\text{X},[{\text{C}}_{\text{p}},\text{C}-{\text{C}}_{\text{p}}])$$

here, ${\text{X}}_{\text{att}}$ corresponds to the first ${\text{C}}_{\text{p}}$ channels of the input, while ${\text{X}}_{\text{res}}$ consists of the remaining $\text{C}-{\text{C}}_{\text{p}}$ channels.

SHSA applies the single-head attention layer only to a portion of the input channels ( ${\text{C}}_{\text{p}}=\text{rC}$), that is, to ${\text{X}}_{\text{att}}$ in order to aggregate spatial features. The remaining channels are kept unchanged. The ratio r is typically set to 1/4.67. The computation is defined in Equation 15 and Equation 16:

(15) $${\tilde{\text{X}}}_{\text{att}}=\text{Attention}({\text{X}}_{\text{att}}{\text{W}}^{\text{Q}},{\text{X}}_{\text{att}}{\text{W}}^{\text{K}},{\text{X}}_{\text{att}}{\text{W}}^{\text{V}})$$

(16) $$\text{Attention}(\text{Q},\text{K},\text{V})=\text{Softmax}(\frac{\text{Q}{\text{K}}^{⊺}}{\sqrt{{\text{d}}_{\text{qk}}}})\text{V}$$

Finally, ${\tilde{\text{X}}}_{\text{att}}$ and ${\text{X}}_{\text{res}}$ are concatenated along the channel dimension and passed through a fully connected layer to obtain the final output. The computation is expressed in Equation 17:

(17) $$\text{SHSA}(\text{X})=\text{Concat}({\tilde{\text{X}}}_{\text{att}},{\text{X}}_{\text{res}}){\text{W}}^{\text{O}}$$

here, $W^{Q}$, $W^{K}$, $W^{V}$, and $W^{O}$ denote the projection weight matrices, $d_{qk}$ represents the dimensionality of the Query and Key vectors, and Concat(·) indicates the concatenation operation. In SHSA, the final projection is performed over all channels rather than being restricted to the initial $C_{p}$ channels, allowing the attention-refined features to be more effectively transmitted to the remaining channels. This strategy avoids the computational redundancy associated with multi-head attention, improves efficiency, and lowers memory access overhead, thereby leading to better overall performance.

### 2.4 Experimental platform and evaluation metrics

Experiments were carried out on a workstation running the 64-bit version of Windows 10. The hardware configuration included a 12-vCPU Intel Xeon Platinum 8255C processor operating at 2.50 GHz and an NVIDIA GeForce RTX 2080 Ti graphics card with 11 GB of memory. All models were implemented and trained in the PyCharm development environment. The software environment was built on a Python 3.8 virtual environment managed with Anaconda3, with PyTorch 2.4.1, CUDA 12.1, and cuDNN 9.1.0 installed. The network was optimized using the Adam optimizer, with the initial learning rate set to 1e-3. The input images were resized to 640 × 640 pixels for training, and the batch size was set to 16.

To comprehensively assess the proposed method, an evaluation system was designed from the aspects of predictive performance, computational cost, and deployment feasibility. Precision, recall, and F1-score were adopted to measure model accuracy. Computational burden was characterized by the parameter count and floating-point operations (FLOPs). In addition, inference speed was used to reflect the method’s real-time applicability in practical deployment scenarios.

#### 2.4.1 Precision

Precision (P) represents the fraction of predicted positive samples that are truly positive. It is used to evaluate the correctness of positive predictions and indicates the model’s ability to reduce false-positive errors. The formula is given in Equation 18:

(18) $$\text{Precision}=\frac{\text{TP}}{\text{TP}+\text{FP}}$$

here, TP refers to the number of truly positive samples that are correctly identified as positive by the model, while FP represents the number of actually negative samples that are mistakenly classified as positive.

#### 2.4.2 Recall

Recall (R) indicates the proportion of real positive samples that are successfully recognized by the model. It reflects the model’s ability to capture target instances comprehensively, and a low recall value suggests that important pathological targets may be missed. It is calculated in Equation 19:

(19) $$\text{Recall}=\frac{\text{TP}}{\text{TP}+\text{FN}}$$

where FN denotes the number of positive samples that are incorrectly classified as negative by the model.

#### 2.4.3 F1-score

The F1-score is defined as the harmonic mean of precision and recall, and it is used to provide a balanced evaluation of these two metrics. This indicator is especially useful for imbalanced datasets, where it can better reflect overall model performance than a single metric alone. A larger F1-score implies a better balance between precision and recall. It is computed in Equation 20:

(20) $$\text{F}1=\frac{2\times \text{Precision}\times \text{Recall}}{\text{Precision}+\text{Recall}}$$

#### 2.4.4 Mean average precision

Mean average precision (mAP) measures the average detection performance of the model over all categories. In practice, it is obtained by averaging the average precision (AP) values across different categories or under specific IoU thresholds. Since it jointly considers precision and recall, mAP provides a more comprehensive assessment of the model’s robustness in detecting objects with different sizes and shapes. A higher IoU value indicates greater overlap between the predicted region and the ground-truth region, corresponding to better segmentation quality. IoU is defined in Equation 21:

(21) $$\text{IoU}=\frac{\left|\text{A}\cap \text{B}\right|}{\left|\text{A}\cup \text{B}\right|}$$

where A denotes the predicted segmentation region and B denotes the ground-truth annotated region:

The calculation of mAP is performed by first obtaining the AP value for each category, where AP corresponds to the area under the Precision–Recall curve. The AP values of all categories are then averaged to obtain mAP, as shown in Equation 22:

(22) $$\text{mAP}=\frac{1}{\text{N}}\sum_{\text{i}=1}^{\text{N}}{\text{AP}}_{\text{i}}$$

where N is the total number of categories and $AP_{i}$is the AP of the i-th category. In particular, mAP@0.5 denotes the mean AP at an IoU threshold of 0.5, meaning that a prediction is regarded as correct when the IoU between the predicted mask and the corresponding ground truth is no less than 0.5. For segmentation tasks, mAP@0.5 is generally more informative than precision or recall alone, especially for evaluating pixel-level prediction quality.

#### 2.4.5 Number of parameters

The number of parameters represents the total amount of trainable weights contained in a model. In deep learning, models with fewer parameters are usually considered more lightweight and may achieve faster inference. This metric is obtained by summing the trainable parameters of all network layers, and it is commonly used to characterize model complexity and storage demand.

#### 2.4.6 Floating point operations

Floating point operations (FLOPs) refer to the total number of floating-point calculations required during a single forward propagation process. This metric is widely used to describe computational complexity and is closely associated with runtime efficiency. In general, a lower FLOPs value indicates reduced computational cost and potentially higher inference efficiency.

#### 2.4.7 Inference speed

Inference speed describes how quickly a model processes an input sample on a given hardware platform and is commonly expressed in frames per second (FPS). The inference time for a single image generally includes three components: pre-processing, forward inference, and post-processing, and is usually measured in milliseconds (ms). FPS represents the number of images that can be processed per second and is an important criterion for evaluating real-time applicability. It is defined in Equation 23:

(23) $$\text{FPS}=\frac{1000}{\text{Preprocess}+\text{Inference}+\text{Postprocess}}$$

### 2.5 Instance-segmentation-based extraction of infection morphological features of cucumber pathogens

**Figure 10:**
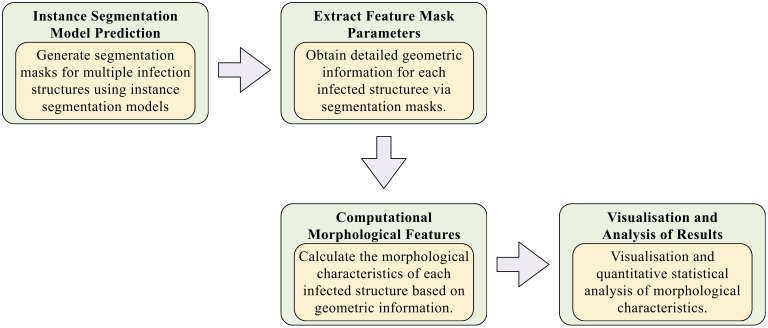
Workflow for extracting morphological features of cucumber pathogen infection based on microscopic image instance segmentation.

This study proposes a complete workflow for extracting morphological features of cucumber pathogen infection by integrating deep-learning-based instance segmentation with plant pathology knowledge. The workflow aims to accurately segment infection structures of cucumber pathogens from microscopic images and further quantify their morphological characteristics, thereby providing a scientific basis for early disease diagnosis and pathological mechanism analysis. The detailed steps of the instance-segmentation-based feature extraction process are illustrated in **Figure 10**.

In the quantitative analysis of cucumber pathogen infection, morphological feature representation is essential for characterizing the infection process, assessing disease severity, and investigating underlying pathological mechanisms. Common morphological indicators of cucumber pathogen infection include area, perimeter, curvature, and spatial distribution density.

#### 2.5.1 Area

Area is a fundamental descriptor used to quantify the size of the pathogen infection region and is one of the key indicators for assessing disease severity. The area of an infection structure can be computed from the size of its two-dimensional projection. The metric is defined in Equation 24:

(24) $$\text{Area}=\sum_{\text{i}=1}^{\text{N}}{\text{δ}}_{\text{i}}\cdot {\text{S}}_{\text{pixel}}$$

here, N is the total number of pixels, ${\text{δ}}_{\text{i}}$ is the binary mask indicator for each pixel (0 or 1), and ${\text{S}}_{\text{pixel}}$ denotes the physical area represented by a single pixel, determined through microscope objective calibration.

#### 2.5.2 Perimeter

Perimeter describes the length of the boundary of a pathogen infection structure. This metric reflects the edge complexity of the infection region and is closely related to the pathogen’s spreading speed and spatial expansion. The perimeter can be computed by summing the distances between consecutive boundary pixels. The metric is defined in Equation 25:

(25) $$\text{Perimeter}=\sum_{\text{i}=1}^{\text{N}}\sqrt{{\left({\text{x}}_{\text{i}+1}-{\text{x}}_{\text{i}}\right)}^{2}+{\left({\text{y}}_{\text{i}+1}-{\text{y}}_{\text{i}}\right)}^{2}}\cdot {\text{L}}_{\text{pixel}}$$

here, $\left({\text{x}}_{\text{i}},{\text{y}}_{\text{i}}\right)$ represent the coordinate sequence of consecutive boundary pixels, and ${\text{L}}_{\text{pixel}}$ denotes the physical edge length of a single pixel.

#### 2.5.3 Curvature

Curvature quantifies the degree of bending along the boundary of a pathogen infection structure and can be used to characterize the surface irregularity of structures such as conidia and sporangia. Larger curvature values may indicate rapid structural expansion or morphological changes. Curvature can be computed using the three-point local curvature method, as defined in Equation 26:

(26) $$\text{Curvature}=\frac{2\cdot |({\text{x}}_{2}-{\text{x}}_{1})({\text{y}}_{3}-{\text{y}}_{1})-({\text{x}}_{3}-{\text{x}}_{1})({\text{y}}_{2}-{\text{y}}_{1})|}{\sqrt{\left[{\left({\text{x}}_{2}-{\text{x}}_{1}\right)}^{2}+{\left({\text{y}}_{2}-{\text{y}}_{1}\right)}^{2}]\cdot [{\left({\text{x}}_{3}-{\text{x}}_{1}\right)}^{2}+{\left({\text{y}}_{3}-{\text{y}}_{1}\right)}^{2}\right]}}$$

here, $({\text{x}}_{1},{\text{y}}_{1}),\;({\text{x}}_{2},{\text{y}}_{2})\text{, }$ and $\left({\text{x}}_{3},{\text{y}}_{3}\right)$ represent the coordinates of three consecutive contour points.

#### 2.5.4 Ellipticity

Ellipticity is an important descriptor of the shape of pathogen infection structures and reflects their directional characteristics. This indicator can be used to analyze the expansion direction and morphological tendencies of pathogen infection. The metric is defined in Equation 27:

(27) $$\text{Ellipticity}=1-\frac{{\text{L}}_{\text{minor}}}{{\text{L}}_{\text{major}}}$$

here, L_minor_ and L_major_ represent the lengths of the minor and major axes, respectively. An ellipticity value close to 0 indicates that the structure is nearly circular, whereas a larger ellipticity suggests that the pathogen infection structure is more elongated.

For the categorical analysis of morphological distributions, the six infection-structure categories were first assigned to two pathogen groups according to their biological origin. The powdery mildew group included PM-Spore, PM-Conidium, and PM-Conidiophore, whereas the downy mildew group included DM-Sporangium, DM-Sporangiophore, and DM-Hyphae. For each infection-structure category, 50 instances were used for morphological statistical analysis. The morphological descriptors, including area, perimeter, curvature, and ellipticity, were compared among categories within each pathogen group.

Specifically, pairwise independent-sample t-tests were performed only among the three infection-structure categories belonging to the same pathogen group. For powdery mildew, the pairwise comparisons included PM-Spore vs. PM-Conidium, PM-Spore vs. PM-Conidiophore, and PM-Conidium vs. PM-Conidiophore. For downy mildew, the pairwise comparisons included DM-Sporangium vs. DM-Sporangiophore, DM-Sporangium vs. DM-Hyphae, and DM-Sporangiophore vs. DM-Hyphae. Statistical significance was defined as p < 0.05. The significance levels were annotated in the corresponding morphological distribution plots using asterisks, where * indicates p < 0.05, ** indicates p < 0.01, and *** indicates p < 0.001. Cross-pathogen pairwise comparisons were not performed because the infection structures from powdery mildew and downy mildew belong to different pathogen taxa and are not strictly biologically equivalent in terms of morphology, developmental role, or infection function.

## 3 Results and discussion

### 3.1 Comparative analysis of SWS-YOLO11n and advanced instance segmentation models

To thoroughly assess the segmentation capability of SWS-YOLO11n on microscopic images of cucumber pathogens, a series of representative state-of-the-art instance segmentation methods were employed for comparative analysis. These methods included two-stage frameworks, such as Mask R-CNN, PointRend, and QueryInst, as well as one-stage frameworks, including YOLACT, YOLOv5n-seg, YOLOv8-seg (n, s, m, l, x), YOLOv9-seg (c, e), YOLO11-seg (n, s, m, l, x), YOLO12-seg (n, s, m, l, x), and YOLO26-seg (n, s, m, l, x). The detailed experimental results are summarized in **Table 2**.

**Table 2:** Performance comparison between SWS-YOLO11n and advanced instance segmentation models.

Model	Box_mAP@.5 (%)	Mask_mAP@.5 (%)	Parameter (M)	FLOPs (G)	Inference speed (FPS)
Mask R-CNN	96.1	90.5	43.99	229.0	19.3
PointRend	95.7	93.7	56.04	176.0	16.9
QueryInst	98.5	97.6	172.12	391.0	18.2
YOLACT	89.9	73.7	34.76	61.9	48.4
YOLOv5n-seg	81.1	67.8	1.89	6.8	24.3
YOLOv8n-seg	95.0	93.0	2.94	10.7	25.4
YOLOv8s-seg	95.3	94.1	10.48	37.4	22.6
YOLOv8m-seg	95.7	94.9	24.59	98.7	21.3
YOLOv8l-seg	96.5	95.7	41.77	201.2	19.7
YOLOv8x-seg	97.6	96.8	65.23	313.9	18.8
YOLOv9c-seg	97.9	96.2	23.46	138.0	20.7
YOLOv9e-seg	98.2	97.3	56.28	228.5	18.9
YOLO11n-seg	96.3	94.4	2.84	10.2	23.9
YOLO11s-seg	96.9	95.1	10.08	35.6	22.1
YOLO11m-seg	97.5	97.7	22.36	123.6	20.8
YOLO11l-seg	98.3	98.2	27.62	142.7	19.2
YOLO11x-seg	98.7	98.4	62.06	319.7	18.5
YOLO12n-seg	96.5	94.7	2.81	9.9	23.8
YOLO12s-seg	97.0	95.2	9.79	33.4	22.3
YOLO12m-seg	97.7	97.8	21.92	115.1	20.7
YOLO12l-seg	98.6	98.4	28.78	137.7	19.7
YOLO12x-seg	98.9	98.5	64.50	308.7	18.8
YOLO26n-seg	96.8	94.9	2.70	9.1	22.7
YOLO26s-seg	97.0	95.4	10.44	34.2	22.0
YOLO26m-seg	97.8	97.9	23.61	121.5	21.7
YOLO26l-seg	98.7	98.7	28.02	139.8	19.9
YOLO26x-seg	99.0	98.9	62.80	313.5	17.4
SWS-YOLO11n	97.4	96.2	2.69	10.0	22.3

As presented in **Table 2**, SWS-YOLO11n delivers better detection and segmentation performance than the two-stage models Mask R-CNN and PointRend, while using only 6.1% and 4.8% of their parameters, respectively. Although its accuracy is slightly below that of QueryInst, the proposed model requires merely 1.6% of QueryInst’s parameter count, indicating a clear advantage in terms of lightweight design. Among the one-stage methods, SWS-YOLO11n performs markedly better than YOLACT and YOLOv5n-seg, exceeds YOLOv8n-seg of similar scale, and achieves results close to those of the much larger YOLOv8x-seg. Within the YOLO11 family, its accuracy is positioned between YOLO11s-seg and YOLO11m-seg, while maintaining evident benefits in model complexity and inference efficiency. These findings confirm the effectiveness of the proposed optimization strategies. Compared with the YOLO12 series models, SWS-YOLO11n outperformed YOLO12s-seg by 0.4 and 1.0 percentage points in Box_mAP@.5 and Mask_mAP@.5, respectively, while its parameter count and computational cost were only 27.48% and 29.94% of those of YOLO12s-seg, demonstrating a significant advantage for deployment. Within the YOLO26 series, SWS-YOLO11n achieves higher detection and segmentation accuracy than both YOLO26n-seg and YOLO26s-seg, while maintaining lower parameter count and computational cost, thereby demonstrating clear advantages for deployment. Overall, SWS-YOLO11n achieves a favorable balance between accuracy, model compactness, and inference speed. It not only outperforms most methods of comparable size and approaches the performance of several larger models, but also preserves a much lower parameter count than the two-stage models and most one-stage alternatives. In addition, its inference efficiency satisfies the real-time demands of microscopic image processing.

### 3.2 Ablation study

To investigate the contributions of the proposed modifications, an ablation experiment was performed. The model was initially trained for 800 epochs without loading pretrained weights. After training, the checkpoint with the best validation performance was selected and tested on the test set. The effects of each modification were then analyzed from the perspectives of detection accuracy, segmentation accuracy, parameter count, computational complexity, and inference speed.

**Figure 11:**
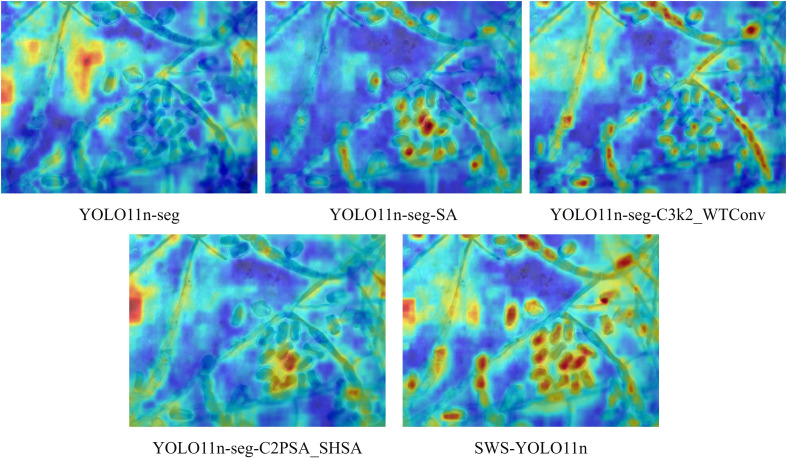
Heatmap visualization of the model.

In the ablation study, five experimental configurations were designed. Group A corresponds to the baseline model YOLO11n-seg. Group B incorporates the Shuffle Attention mechanism into the baseline to capture global contextual information, improve recognition of pathogens of varying sizes, and enhance detail and instance-level segmentation accuracy. Based on Group A, Group C replaces the C3k2 module in the baseline with the C3k2_WTConv module to improve multi-scale perception of pathogen structures; by enhancing high-frequency features, this modification strengthens the extraction of fine details and boundary information, thereby reducing mis-segmentation. Based on Group A, Group D replaces the C2PSA module in the baseline with the C2PSA_SHSA module to reduce computational redundancy and memory access cost while preserving both global and local feature interactions, resulting in improved segmentation efficiency and accuracy. Group E represents the final improved model, combining all enhancements from Groups B, C, and D on top of the baseline. Gradient-weighted Class Activation Mapping (Grad-CAM) is a gradient-based class activation visualization technique that highlights the key regions of interest used by a convolutional neural network during decision-making, thereby revealing the model’s semantic sensitivity to the target class (Vinogradova et al., 2020). By generating heatmaps, Grad-CAM visualizes the spatial regions that contribute most to the model’s predictions, providing an interpretable explanation of the decision process. **Figure 11** presents the Grad-CAM heatmap visualizations for the five experimental configurations.

Analysis of the heatmaps reveals that the baseline model YOLO11n-seg struggles to accurately recognize pathogen infection structures under complex backgrounds, often misidentifying large background regions as highly salient areas. In Group B, the introduction of the SA attention mechanism significantly enhances the model’s ability to detect small targets such as conidia and conidiophores, leading to substantially improved focus on fine-scale infection structures. In Group C, integrating WTConv into the C3k2 module strengthens the model’s capacity to extract both detailed and global features, resulting in increased sensitivity to infection structures of various sizes—including conidia and conidiophores—and achieving a more balanced attention distribution across multi-scale targets. In Group D, incorporating the SHSA attention mechanism into the C2PSA module reduces computational redundancy and memory access cost while simultaneously improving attention to regions containing conidiophores, demonstrating its advantage in small-target learning. Group E, which integrates all three improvements, shows consistently high attention sensitivity to all infection structures even under challenging background conditions. These results indicate that the proposed improvements play a critical role in guiding the model’s decision-making process and effectively enhance the accuracy of pathogen infection structure recognition.

To further assess the effectiveness of the proposed modifications, **Table 3** summarizes the results of five ablation settings on the test set. In particular, ① denotes the introduction of the SA mechanism, ② denotes substituting the C3k2 module with C3k2_WTConv, and ③ denotes replacing the C2PSA module with C2PSA_SHSA.

**Table 3:** Performance comparison of the ablation experiments.

Experiment	Model	Box_mAP@.5 (%)	Mask_mAP@.5 (%)	Parameter (M)	FLOPs (G)	Inference speed (FPS)
A	YOLO11n-seg	96.3	94.4	2.84	10.2	23.9
B	YOLO11n-seg+①	97.1	95.4	2.84	10.2	21.8
C	YOLO11n-seg+②	96.8	95.7	2.72	10.1	21.6
D	YOLO11n-seg+③	95.8	95.5	2.78	10.1	24.1
E	YOLO11n-seg+①②③	97.4	96.2	2.69	10.0	22.3

As indicated in **Table 3**, after inserting the SA mechanism between the Backbone and Neck, Group B improves detection mAP@0.5 by 0.8% and segmentation mAP@0.5 by 1.0%, with almost no change in parameter count or FLOPs. This suggests that the SA module can enhance both detection and segmentation performance without introducing extra model complexity. For Group C, replacing C3k2 with C3k2_WTConv leads to a 0.5% increase in detection mAP@0.5 and a larger 1.3% gain in segmentation mAP@0.5. This improvement can be attributed to the stronger capability of WTConv to capture both local details and global contextual information, which benefits segmentation of all infection structures. In addition, owing to its more parameter-efficient design, the parameter count is reduced by 0.12 M and FLOPs are lowered by 0.1 G. In Group D, substituting C2PSA with C2PSA_SHSA decreases the number of parameters by 0.06 M and FLOPs by 0.1 G, while the inference speed is improved by 0.2 FPS. These results indicate that SHSA can effectively reduce computational redundancy and memory access overhead, thereby improving efficiency. Although detection mAP@0.5 drops by 0.5%, segmentation mAP@0.5 still rises by 1.1%, implying that modeling long-range spatial dependencies helps strengthen feature fusion and provides a better trade-off between accuracy and efficiency. Group E combines all the improvements introduced in Groups B, C, and D. The resulting model obtains gains of 1.1% in detection mAP@0.5 and 1.8% in segmentation mAP@0.5, while reducing the parameter count by 0.15 M and FLOPs by 0.2 G. Although the inference speed shows a slight decline, with FPS decreasing by 1.6, the overall results demonstrate that the proposed modifications can simultaneously improve model performance and reduce model complexity.

To analyze the impact of the proposed improvements on detecting and segmenting different infection structures, **Table 4** reports the mAP@0.5 values of bounding boxes for each structure before and after model enhancement. **Table 5** reports the corresponding mAP@0.5 values of masks for each structure before and after model enhancement. As shown in the tables, the SWS-YOLO11n model achieves performance gains across all six infection-structure categories compared with the baseline model. The improvement is particularly notable for hyphae, which exhibit more complex morphology, with detection mAP@0.5 increasing by 7.4% and segmentation mAP@0.5 increasing by 2.6%.

**Table 4:** Detection performance (mAP@0.5) on different infection structures before and after model improvement.

Model	Powdery mildew			Downy mildew		
	Spore	Conidia	Conidiophore	Sporangia	Sporangiophore	Hyphae
YOLO11n	97.4	98.3	98.4	98.5	98.1	82.3
SWS-YOLO11n	98.4	98.4	98.5	98.5	98.5	89.7

**Table 5:** Segmentation performance (mAP@0.5) on different infection structures before and after model enhancement.

Model	Powdery mildew			Downy mildew		
	Spore	Conidia	Conidiophore	Sporangia	Sporangiophore	Hyphae
YOLO11n	96.3	97.3	78.5	97.5	97.1	82.4
SWS-YOLO11n	97.5	97.3	84.3	97.5	97.5	85.0

**Figure 12:**
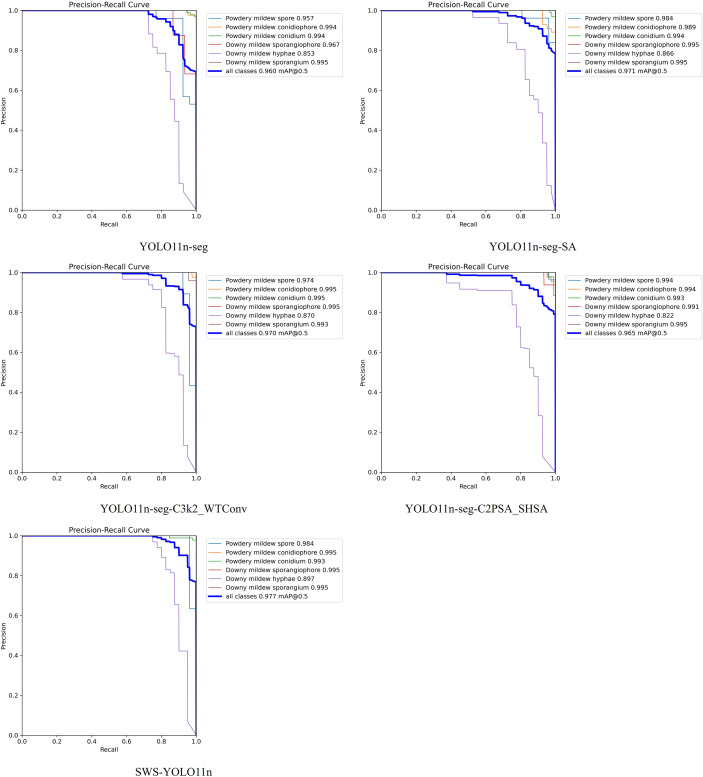
Box-PR curves of the ablation experiments.

**Figure 13:**
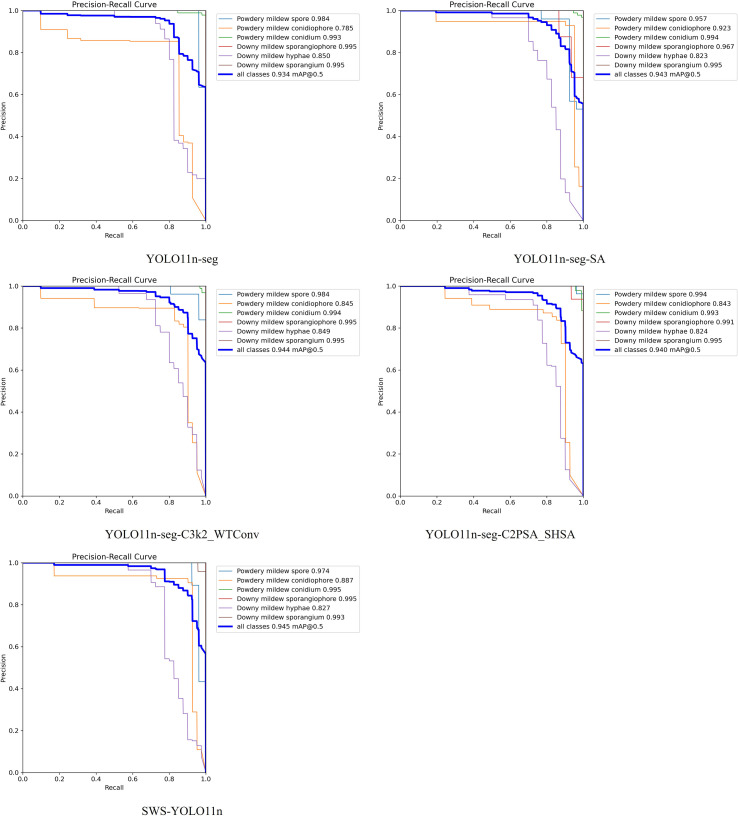
Mask-PR curves of the ablation experiments.

**Figures 12**, **13** illustrate the PR curves for different infection structures before and after the model improvements. Analysis of these results shows that the effectiveness of each enhancement varies across infection-structure types. In terms of detection performance, introducing the SA attention mechanism increases the AP for powdery mildew conidia, downy mildew sporangiophores, and downy mildew hyphae by 2.7%, 2.8%, and 1.3%, respectively. Incorporating WTConv improves the AP for powdery mildew conidia, downy mildew sporangiophores, and downy mildew hyphae by 1.7%, 2.8%, and 1.7%, respectively. Adding the SHSA attention mechanism further increases the AP for powdery mildew conidia and downy mildew sporangiophores by 3.7% and 2.4%, respectively. Ultimately, the fully improved model achieves AP gains of 2.7%, 2.8%, and 4.4% for powdery mildew conidia, downy mildew sporangiophores, and downy mildew hyphae, respectively, with an overall improvement of 1.7% in mean detection precision.

In terms of segmentation performance, the three enhancement modules increase the AP of powdery mildew conidiophores by 13.8%, 6.0%, and 5.8%, respectively. The final integrated model achieves a 10.2% improvement in the AP of powdery mildew conidiophores and a 1.1% improvement in the overall mean segmentation precision.

**Figure 14:**
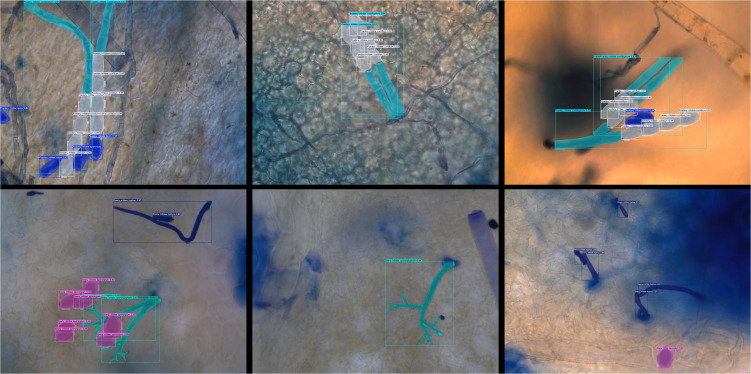
Instance segmentation results of the improved model.

Using the trained model, predictions were performed on real microscopic images, and the results are shown in **Figure 14**. As illustrated by the segmentation outputs, the SWS-YOLO11n model is able to accurately segment all six types of infection structures even under complex background interference. The model maintains high detection accuracy despite large differences in target size (e.g., conidia vs. conidiophores) or similar yet distinct structural complexity (e.g., hyphae vs. sporangiophores). In all cases, the prediction confidence exceeds 90%. This performance advantage provides a high-fidelity segmentation foundation for establishing a quantitative morphological characterization framework across multiple infection-structure categories.

### 3.3 Analysis of morphological feature extraction performance

Regression analysis is a widely used statistical approach for exploring relationships between variables. To rigorously validate the model’s accuracy in extracting morphological features, after completing the segmentation of infection structures in all test-set images, this study performed stratified random sampling from the multiple categories of infection-structure masks predicted by the model. Specifically, 50 instances were randomly selected from each of the six infection-structure categories, yielding a total of 300 samples. These sampled masks were then matched with their corresponding manual annotations according to their spatial positions in the images, and the extraction accuracy of morphological descriptors, including area, perimeter, curvature, and ellipticity, was subsequently evaluated. The coefficient of determination (R²), root mean squared error (RMSE), and mean absolute error (MAE) were used to assess the accuracy of morphological feature extraction. In addition, Pearson’s correlation coefficient (r) and its significance level (p value) were used to comprehensively evaluate the agreement and error characteristics between the predicted values and the reference ground-truth values, thereby strengthening the statistical support of the analysis.

**Figure 15:**
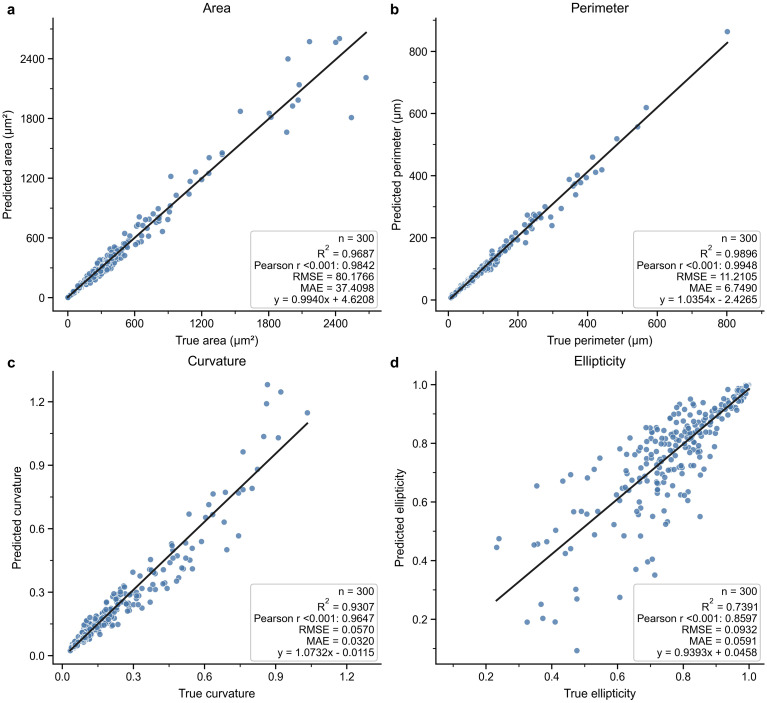
Regression curves of morphological feature indices. **(a)** Area. **(b)** Perimeter. **(c)** Curvature. **(d)** Ellipticity.

**Figure 15** presents the regression relationships between the predicted values and the reference ground-truth values for four morphological indicators, namely area, perimeter, curvature, and ellipticity, while **Table 6** further summarizes the corresponding statistical results. Overall, all four indicators showed statistically significant correlations between predicted and reference values (p < 0.001), indicating that the instance-segmentation-based morphological feature extraction method was able to recover the two-dimensional morphological information of cucumber pathogen infection structures with good fidelity. Among these indicators, area showed good fitting performance, with an R² of 0.9687, a Pearson’s r of 0.9842, and RMSE and MAE values of 80.1766 and 37.4098, respectively. As shown in **Figure 15a**, most sample points are closely distributed around the regression line, indicating that the model provides generally stable estimates of area; only a small number of samples in the high-value region deviate markedly from the regression line, suggesting that area prediction may still be affected when the infection structures are relatively large or morphologically more complex. Perimeter exhibited the best predictive performance, with an R² of 0.9896, a Pearson’s r of 0.9948, and RMSE and MAE values of 11.2105 and 6.7490, respectively. In **Figure 15b**, the scatter points are the most tightly clustered and generally aligned along the regression line, demonstrating that the model can characterize boundary length with high consistency and accuracy and can therefore accurately reflect changes in the contours of infection structures. For curvature, the R² was 0.9307, the Pearson’s r was 0.9647, and the RMSE and MAE were 0.0570 and 0.0320, respectively. Although the dispersion of the curvature data is slightly greater than that observed for area and perimeter, most points in **Figure 15c** still remain close to the regression line, indicating that the model retains good representational capability for this relatively complex morphological characteristic describing boundary bending. In contrast, the predictive performance for ellipticity was clearly weaker than that for the other three indicators, with an R² of only 0.7391, a Pearson’s r of 0.8597, and RMSE and MAE values of 0.0932 and 0.0591, respectively. As shown in **Figure 15d**, the scatter points for ellipticity are more widely dispersed, and the deviations from the regression line are more evident, particularly in the low- to mid-value range, indicating that the model is relatively less stable and less accurate in predicting this indicator. Overall, the model achieved high goodness of fit and good error control for area, perimeter, and curvature, whereas the prediction accuracy for ellipticity was comparatively lower. This suggests that ellipticity may be more susceptible to the combined effects of infection-structure type, boundary segmentation error, and morphological scale differences, and therefore warrants further category-specific accuracy analysis and targeted methodological improvement.

**Table 6:** Error analysis of morphological feature indices.

Morphological feature	R^2^	r	p	RMSE	MAE
Area	0.9687	0.9842	3.216840e-226	80.1766	37.4098
Perimeter	0.9896	0.9948	1.021057e-297	11.2105	6.7490
Curvature	0.9307	0.9647	8.940684e-175	0.0570	0.0320
Ellipticity	0.7391	0.8597	6.143401e-89	0.0932	0.0591

Further analysis indicated that the lower prediction accuracy of ellipticity, compared with the other morphological indicators, was mainly related to its calculation method and the structural characteristics of the target objects. Unlike area and perimeter, which are primarily derived from the accumulation of the overall region or the entire boundary, ellipticity is calculated as one minus the ratio of the minor-axis length to the major-axis length of the fitted ellipse. Therefore, it is more sensitive to local boundary perturbations. For small-scale and nearly circular structures, such as spores and sporangia, the major and minor axes are relatively close in value. Slight boundary segmentation deviations, edge blurring, or local noise may therefore cause noticeable changes in the estimated major and minor axes, and these changes can be further amplified during the ratio calculation. In contrast, elongated structures have a more pronounced difference between the major and minor axes and stronger directionality, making their ellipticity relatively less sensitive to local contour errors. Thus, the lower accuracy of ellipticity does not necessarily indicate that the model fails to capture morphological differences; rather, it suggests that this indicator imposes higher requirements on segmentation boundary quality and the stability of axial estimation for small, nearly circular structures.

**Figure 16:**
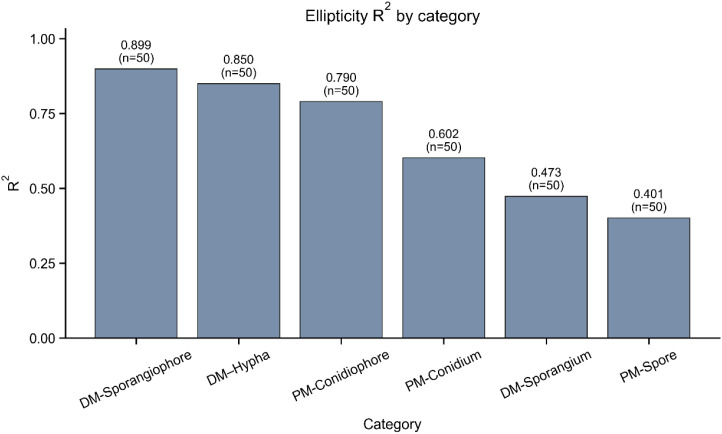
Comparison of R^2^ values for ellipticity prediction across different infection structure categories.

To further investigate the relatively lower overall prediction accuracy of the ellipticity parameter, the model performance was analyzed in a more fine-grained manner according to infection-structure category, as shown in **Figure 16**. Clear differences in ellipticity prediction accuracy were observed among the different categories. The best performance was achieved for downy mildew sporangiophores (DM-Sporangiophore), with an R² of 0.899, followed by downy mildew hyphae (DM-Hypha, R² = 0.850) and powdery mildew conidiophores (PM-Conidiophore, R² = 0.790). Powdery mildew conidia that had not yet detached (PM-Conidium) showed intermediate prediction accuracy (R² = 0.602), whereas downy mildew sporangia (DM-Sporangium, R² = 0.473) and detached powdery mildew spores (PM-Spore, R² = 0.401) exhibited relatively poor predictive performance. These results indicate that the model is more capable of characterizing the ellipticity of stalk-like structures that are slender, strongly directional, and morphologically elongated, whereas prediction errors are more pronounced for spore/sporangium-like structures that are smaller in size, closer to circular in shape, and more sensitive to boundary variations.

The above differences may be attributed to the following reasons. On the one hand, spore- and sporangium-like targets are relatively small in microscopic images, with blurred boundaries and weak local contrast. Even slight segmentation deviations may cause substantial fluctuations in the estimated major and minor axes, thereby amplifying errors in ellipticity calculation. On the other hand, ellipticity itself is a compound descriptor calculated as one minus the ratio of the minor-axis length to the major-axis length of the fitted ellipse, making it inherently more sensitive to local perturbations along the contour boundary. Therefore, the relatively low prediction accuracy of ellipticity cannot be explained solely by its constrained value range, but is also closely related to the morphological complexity of different infection structures and their sensitivity to segmentation errors.

From the perspective of biological interpretation, this result suggests that the reliability of ellipticity varies among different infection structures. For elongated and directionally distinct structures, such as DM-Sporangiophore, DM-Hyphae, and PM-Conidiophore, ellipticity can relatively stability reflect their degree of elongation and directional characteristics, and can therefore be used to assist in understanding their functional differentiation in support, extension, and spore production. In contrast, for nearly circular dispersal structures, such as PM-Spore and DM-Sporangium, subtle differences in ellipticity at the individual-instance level should not be overinterpreted as clear biological differences because of the relatively larger prediction error. For these structures, ellipticity is more suitable as an auxiliary indicator of category-level morphological trends and should be interpreted together with more stable indicators, such as area, perimeter, and curvature. In other words, the relatively lower accuracy of ellipticity does not change the overall conclusion of this study that different infection structures exhibit morphological differentiation, but it does require a more cautious interpretation of ellipticity differences in small, nearly circular structures.

**Figure 17:**
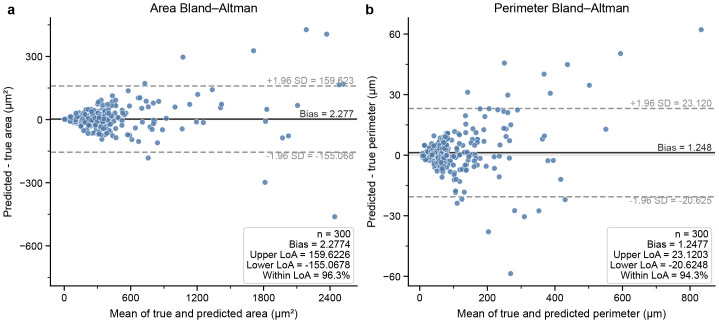
Bland–Altman analysis of area and perimeter.

To further evaluate the consistency between the model-predicted and manually referenced morphological measurements, Bland–Altman analyses were conducted for area and perimeter, and the results are presented in **Figure 17**. The results showed that the mean bias for area prediction was 2.2774 μm², with 95% limits of agreement (LoA) ranging from −155.0678 μm² to 159.6226 μm². A total of 96.3% of the sample points fell within the limits of agreement, indicating good agreement between the model estimates and manual measurements for area. For perimeter prediction, the mean bias was 1.2477 μm, and the 95% limits of agreement ranged from −20.6248 μm to 23.1203 μm. A total of 94.3% of the sample points fell within the limits of agreement, indicating generally good agreement overall. Overall, the Bland–Altman analysis further confirmed the reliability of the model in predicting area and perimeter. Among the two morphological parameters, area showed more stable agreement.

To further validate the biological plausibility of the extracted morphological values, the present study compared the measurements obtained from the segmented masks of representative infection structures with morphological values reported in previous plant pathology studies. It should be noted that the existing literature generally reports the length, width, and length-to-width ratio of conidia or sporangia, whereas mask-based statistical descriptors such as area, perimeter, and curvature are rarely reported directly. Previous studies have shown that the conidia of powdery mildew (*Podosphaera xanthii*) are approximately 26–40 μm × 12–18 μm in size, and the conidiophores are approximately 76–200 μm × 8–14 μm (Uchida et al., 2009). Another study reported conidial dimensions of 30.5 ± 3.1 μm × 17.7 ± 2.7 μm (Sletova et al., 2024). For downy mildew (*Pseudoperonospora cubensis*), one study reported sporangial dimensions of 19.2–26.4 μm × 14.1–18.5 μm, with a length-to-width ratio of 1.26–1.54, and sporangiophore lengths of 259–449 μm (Runge et al., 2012). Another study reported sporangial dimensions of 22–38 μm × 15–22 μm, a length-to-width ratio of 1.4–1.7, and sporangiophore lengths of 120–480 μm (Young-Joon et al., 2005). Compared with these published reports, the morphological values extracted in this study generally fall within similar ranges and are broadly consistent with previous findings, indicating that the morphological parameters derived from the segmented masks are biologically reasonable from a plant pathology perspective.

The quantitative morphological results obtained in this study may provide practical support for disease prevention and control. Specifically, microscopic indices such as area, perimeter, curvature, and ellipticity can help identify pathogen developmental stages and subtle morphological transitions before obvious macroscopic symptoms appear, thereby contributing to early disease diagnosis and the determination of critical control windows. In addition, these quantitative traits may serve as auxiliary indicators for evaluating pathogen developmental activity and disease severity, offering more objective evidence for disease monitoring and epidemic trend analysis. Furthermore, changes in the morphology of infection structures under different treatment conditions may help assess the inhibitory effects of fungicides or other control measures on pathogen development, thus supporting fungicide screening, control-effect evaluation, and precision disease management.

### 3.4 Morphological distribution of cucumber pathogen infection structures

**Figure 18:**
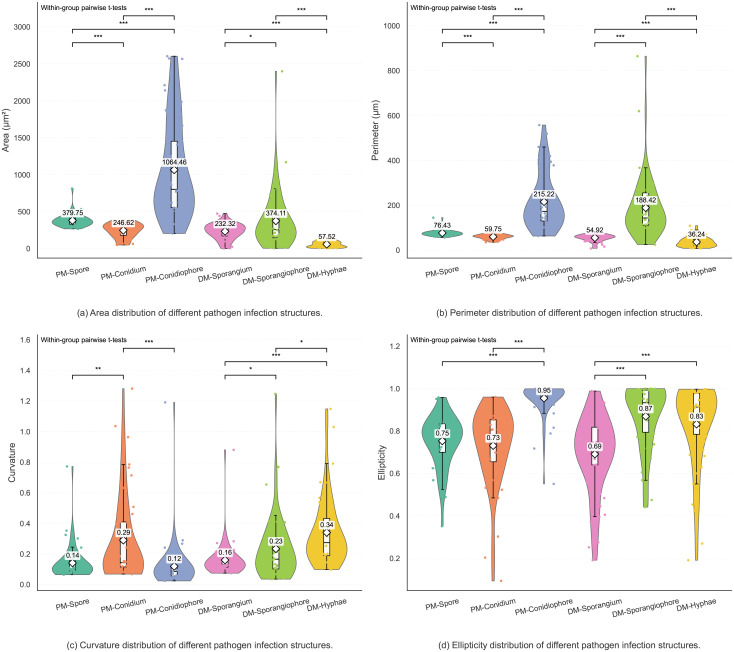
Morphological distribution of cucumber pathogen infection structures. Asterisks indicate the significance levels of within-group pairwise t-tests: * indicates p < 0.05, ** indicates p < 0.01, and *** indicates p < 0.001.

To further enhance the plant pathological interpretability of this study, in addition to validating model accuracy, we further performed a categorical statistical analysis of the morphological distributions of different infection structures of cucumber powdery mildew and downy mildew, as shown in **Table 7** and **Figure 18**. For each morphological parameter, 50 instances were analyzed for each infection-structure category. Pairwise t-tests were conducted only among the three infection-structure categories within the same pathogen group, including PM-Spore, PM-Conidium, and PM-Conidiophore for powdery mildew, and DM-Sporangium, DM-Sporangiophore, and DM-Hyphae for downy mildew. Cross-pathogen statistical comparisons were not performed because the corresponding structures belong to different pathogen taxa and have distinct biological attributes. In **Figure 18**, white diamond markers indicate the mean values of each category, and asterisks indicate statistically significant within-pathogen pairwise differences. This section discusses the morphological differentiation patterns within each pathogen and further provides a descriptive comparison of functionally corresponding infection structures between the two pathogens, with the aim of exploring the potential biological significance reflected by these morphological differences.

**Table 7:** Descriptive statistics of morphological parameters for different cucumber pathogen infection structures.

Shape indicators	Descriptive statistics	PM-Spore	PM-Conidium	PM-Conidiophore	DM-Sporangium	DM-Sporangiophore	DM-Hyphae
Area (μm^2^)	Mean	379.75	246.62	1064.46	232.32	374.11	57.52
	Standard Deviation	87.91	98.58	667.98	106.80	392.98	42.95
	Skewness	2.55	-0.53	0.87	-0.35	3.16	0.46
	Kurtosis	10.98	-0.67	-0.31	-0.08	13.83	-0.99
Perimeter (μm)	Mean	76.43	59.75	215.22	54.92	188.42	36.24
	Standard Deviation	15.42	11.11	123.45	16.23	140.69	22.02
	Skewness	2.53	-0.37	1.10	-1.20	2.92	1.03
	Kurtosis	8.40	-0.38	0.43	1.58	11.32	1.23
Curvature	Mean	0.14	0.29	0.12	0.16	0.23	0.34
	Standard Deviation	0.11	0.28	0.17	0.12	0.21	0.22
	Skewness	3.98	1.78	5.80	5.10	2.73	1.84
	Kurtosis	19.96	2.77	37.47	30.86	10.25	3.84
Ellipticity	Mean	0.75	0.73	0.95	0.69	0.87	0.83
	Standard Deviation	0.12	0.18	0.08	0.18	0.16	0.20
	Skewness	-1.08	-1.64	-3.40	-0.91	-1.33	-1.66
	Kurtosis	1.60	3.20	13.30	0.61	0.96	2.28

In powdery mildew, different infection structures exhibited a relatively clear functional morphological division. Among the three powdery mildew structures, the conidiophore (PM-Conidiophore) had the largest mean area and perimeter, reaching 1064.46 μm² and 215.22 μm, respectively, and also showed the highest mean ellipticity of 0.95. The within-pathogen pairwise t-tests showed that PM-Conidiophore differed significantly from the two conidial categories in area, perimeter, and ellipticity, indicating that this structure is overall more elongated, more directionally oriented, and more extended in contour. This characteristic is consistent with the functional role of the conidiophore as a structure for conidium production and support, as it requires a relatively large extension scale and strong directionality to support the formation and release of conidia. In contrast, the two types of powdery mildew conidia were overall more compact. The detached conidium (PM-Spore) had a mean area and perimeter of 379.75 μm² and 76.43 μm, respectively, both higher than those of the attached conidium (PM-Conidium), which were 246.62 μm² and 59.75 μm. The pairwise t-tests further indicated significant differences between these two conidial categories in size-related parameters, supporting the presence of morphological differences between the detached and attached states. However, their mean ellipticity values were relatively close, 0.75 and 0.73, respectively, suggesting that both belong to relatively compact dispersal structures, although certain differences in size and boundary morphology still exist between the two states. In addition, PM-Conidium showed a higher mean curvature of 0.29 than PM-Spore and PM-Conidiophore, indicating more pronounced boundary variation and a more irregular morphology. Overall, powdery mildew exhibited an internal structural differentiation pattern in which conidiophores are mainly responsible for support and conidium production, whereas conidia mainly serve for dispersal, and this differentiation can be quantitatively characterized by morphological parameters.

In downy mildew, different infection structures likewise showed a clear hierarchical differentiation. The branched sporangiophore (DM-Sporangiophore) had relatively large mean area and perimeter values of 374.11 μm² and 188.42 μm, respectively, together with a relatively high mean ellipticity of 0.87. The within-pathogen pairwise t-tests showed significant differences among the three downy mildew structures for several major size- and shape-related parameters, particularly perimeter and ellipticity, indicating that the branched sporangiophore is distinctly elongated and structurally differentiated from the more compact sporangium and the slender unbranched hypha-like structure. This is consistent with its role as a reproductive supporting structure for the formation and bearing of sporangia. By contrast, the unbranched sporangiophore (DM-Hyphae) showed the lowest mean area and perimeter, only 57.52 μm² and 36.24 μm, respectively, but the highest mean curvature of 0.34, suggesting that this structure more closely represents a slender, elongating growth state before obvious branching occurs, with greater boundary bending and exploratory growth characteristics. The sporangium (DM-Sporangium), on the other hand, exhibited a relatively small scale and a more rounded contour, with a mean area of 232.32 μm², a mean perimeter of 54.92 μm, and the lowest mean ellipticity of 0.69, indicating that as a dispersal unit, it is morphologically more compact rather than elongated. These findings suggest that downy mildew possesses an internal morphological hierarchy consisting of elongating growth structures, branched supporting structures, and dispersal structures, and that this hierarchy is broadly consistent with the distinct biological roles of these structures during infection and reproduction.

A descriptive comparison between powdery mildew and downy mildew further suggests that, although the two pathogens belong to fungi and oomycetes, respectively, and differ in infection mode and life history, certain functionally corresponding structures exhibit similar morphological tendencies. In general, stalk-like structures involved in support, extension, or spore production, such as powdery mildew conidiophores and branched or unbranched downy mildew sporangiophores, tend to have higher ellipticity and stronger directionality, whereas spore- or sporangium-type structures involved in dispersal are overall more compact and closer to circular in shape. It should be noted that the statistical significance tests in this section were restricted to within-pathogen comparisons, whereas cross-pathogen comparisons were interpreted descriptively because of the biological non-equivalence of the corresponding infection structures. These results indicate that the morphological indices extracted in this study can not only be used to evaluate model accuracy, but can also reveal structurally differentiated features with plant pathological significance at the level of different infection structures, thereby providing a quantitative basis for understanding the structural adaptation of the two pathogens in processes such as extension, reproduction, and dispersal.

### 3.5 Directions for further research

#### 3.5.1 Improving generalization and robustness of the algorithm

Although this study achieved promising results in instance segmentation and morphological feature extraction of pathogen infection structures, the generalization and robustness of the proposed method can be further strengthened by incorporating more diverse sample sources and validation scenarios. The data used in this study were obtained from microscopic samples prepared within a relatively consistent experimental and institutional setting. Specifically, the pathogen source originated from cucumber leaves collected in the greenhouse of the Innovation Base of the Tianjin Academy of Agricultural Sciences, and subsequent sample acquisition, slide preparation, and microscopic imaging were conducted under controlled and standardized conditions. This strategy helped reduce interference from contaminating microorganisms, leaf-surface impurities, and complex backgrounds, thereby improving image quality and the reliability of infection-structure segmentation and morphological parameter extraction. However, because the current dataset mainly reflects samples prepared under similar experimental conditions, further validation using samples from broader sources would be valuable for evaluating the applicability of the method across different production and laboratory scenarios.

It should be noted that the pathogen source used in this study was directly obtained from cucumber leaves grown under greenhouse production conditions, and the host background was consistent with the intended application scenario. Therefore, the microscopic morphology of the infection structures obtained in this study has a certain degree of practical representativeness for similar greenhouse production conditions. Nevertheless, such representativeness mainly reflects consistency in the source scenario and does not fully replace external validation across broader conditions. In future work, the generalization ability of both the model and the morphological reference values will be further evaluated from three aspects. First, external validation sets including *in situ* greenhouse samples and naturally infected field samples will be constructed to examine the robustness of the model under more complex background conditions. Second, samples from different cucumber cultivars, growth stages, and disease-development conditions will be incorporated to assess the influence of host-background variation on the morphological parameters of infection structures. Third, data generated under different laboratory sample-preparation and imaging conditions will be introduced to conduct cross-laboratory validation. We expect that incorporating more diverse microscopic image samples into the model training and validation pipeline will further improve the accuracy of morphological feature extraction for pathogen infection structures and enhance the applicability of the proposed method in practical production scenarios.

#### 3.5.2 Improving the accuracy of morphological feature extraction for specific infection structures

Although this study achieved high accuracy in predicting morphological indicators such as area, perimeter, and curvature, there is still room for improvement in the morphological characterization of small infection structures such as conidia and sporangia, particularly for the ellipticity descriptor. Because these structures are small in size, often have blurred boundaries, and are morphologically close to circular, even slight segmentation deviations may cause noticeable fluctuations in the estimated major and minor axes, thereby amplifying errors in ellipticity calculation. Future work may further improve the quantitative accuracy of morphological characterization for such small infection structures in several aspects. First, at the data acquisition level, optimizing microscopic imaging conditions, staining quality, and slide preparation procedures may improve image contrast and boundary clarity for small-scale targets, thereby reducing noise at the source. Second, at the data annotation level, more refined labeling of small infection-structure samples, including conidia and sporangia, combined with expert review by plant pathologists, may further improve the accuracy and consistency of annotation boundaries. Third, at the model level, multi-scale feature fusion strategies, boundary-aware branches, or contour-constrained loss functions tailored for small targets may be introduced to enhance the model’s ability to capture subtle edges and near-circular contours. Finally, at the morphological measurement level, sub-pixel contour fitting, boundary smoothing, and more robust major- and minor-axis estimation methods may be incorporated to reduce ellipticity errors caused by pixel-based representation and local boundary perturbations, thereby further improving the reliability of quantitative morphological analysis for small infection structures.

## 4 Conclusion

This study investigates the automated analysis of microscopic images of cucumber powdery mildew and downy mildew. To overcome the limitations of conventional biological experiments in high-throughput analysis and quantitative characterization, an *in situ* stained microscopic image dataset was established, and an instance segmentation model named SWS-YOLO11n was proposed to accurately detect and segment the key infection structures of cucumber pathogens. Based on these segmentation results, quantitative extraction of morphological features—including perimeter, area, curvature, and ellipticity—was performed, making it possible to reveal the developmental characteristics and functional differentiation of different pathogen infection structures from the perspective of “morphology–function” adaptability. Experimental results show that SWS-YOLO11n achieves a detection mAP@0.5 of 97.4% and a segmentation mAP@0.5 of 96.2% on the test set, with a model size of only 5.6 MB. The extraction accuracy of morphological features reaches R² > 0.90 for most indicators, demonstrating both high accuracy and lightweight characteristics suitable for deployment in greenhouses and edge devices. In addition, the morphological distribution analysis showed that stalk-like structures associated with support and extension generally exhibited larger size and higher ellipticity, whereas dispersal-related structures were overall more compact and closer to circular in shape. These results indicate that the extracted morphological parameters can reflect biologically meaningful structural differentiation among infection structures. Overall, the proposed method not only enables high-throughput quantitative characterization of cucumber pathogen infection structures, but also provides quantitative support for understanding their developmental characteristics and functional differentiation.

Future work may further deepen this line of research in two aspects. First, the current two-dimensional static morphological analysis based on microscopic images can be extended to three-dimensional reconstruction and spatiotemporal tracking, so as to continuously characterize the development and morphological evolution of pathogen infection structures across successive infection stages. Second, further studies may explore the relationships between quantitative indices such as area, perimeter, curvature, and ellipticity and pathogen infectivity, disease severity, and responses to control measures, thus enhancing the practical value of morphological phenotyping for disease monitoring, early warning, and precision management.

## Data Availability

The raw data supporting the conclusions of this article will be made available by the authors, without undue reservation.

## References

[B1] Alomran M. M. Noman A. Aqeel M. Khalid N. Maqsood M. F. Akhter N. . (2023). Relative biochemical and physiological docking of cucumber varieties for supporting innate immunity against Podosphaera xanthii. Microb. Pathogen. 184, 106359. doi: 10.1016/j.micpath.2023.106359 37716624

[B2] Bahmani R. Rathor P. More P. Prithiviraj B. (2025). Synergistic activation of grapevine defense mechanisms against downy mildew by Ascophyllum nodosum extract and Pseudomonas fluorescens CHA0. Front. Plant Sci. 16, 1568426. doi: 10.3389/fpls.2025.1568426 40538871 PMC12176806

[B3] Bangun M. B. Herdiyeni Y. Herliyana E. N. (2016). Morphological feature extraction of Jabon’s leaf seedling pathogen using microscopic image. TELKOMNIKA (Telecommunication Computing Electron. Control) 14, 254–261. doi: 10.12928/telkomnika.v14i1.2486

[B4] Finder S. E. Amoyal R. Treister E. Freifeld O. (2024). “ Wavelet convolutions for large receptive fields”, in: European conference on computer vision (Cham: Springer Nature Switzerland), 363–380. doi: 10.1007/978-3-031-72949-2_21

[B5] Fujieda S. Takayama K. Hachisuka T. (2018). “ Wavelet convolutional neural networks,” in Arxiv Preprint Arxiv:1805.08620 (Ithaca, NY, USA:: Cornell University). doi: 10.48550/arXiv.1805.08620

[B6] Han Z. Qiao C. Qin Y. Chen F. Li Y. Zhang L. . (2026). Quantitative characterization of lesion features and analysis of coupled stress effects of mixed infection by downy mildew and powdery mildew pathogens in cucumbers based on instance segmentation. Comput. Electron. Agric. 242, 111286. doi: 10.1016/j.compag.2025.111286 38826717

[B7] Hsiao T. Y. Chang Y. C. Chou H. H. Chiu C. T. (2019). Filter-based deep-compression with global average pooling for convolutional networks. J. Syst. Archit. 95, 9–18. doi: 10.1016/j.sysarc.2019.02.008 38826717

[B8] Ishii H. Fraaije B. A. Sugiyama T. Noguchi K. Nishimura K. Takeda T. . (2001). Occurrence and molecular characterization of strobilurin resistance in cucumber powdery mildew and downy mildew. Phytopathology 91, 1166–1171. doi: 10.1094/PHYTO.2001.91.12.1166 18943331

[B9] Javidan S. M. Banakar A. Vakilian K. A. Ampatzidis Y. Rahnama K. (2024). Diagnosing the spores of tomato fungal diseases using microscopic image processing and machine learning. Multimedia Tools Appl. 83, 67283–67301. doi: 10.1007/s11042-024-18214-y 30311153

[B10] Jiang P. Ergu D. Liu F. Cai Y. Ma B. (2022). A review of Yolo algorithm developments. Proc. Comput. Sci. 199, 1066–1073. doi: 10.1016/j.procs.2022.01.135 38826717

[B11] Li K. Zhu X. Qiao C. Zhang L. Gao W. Wang Y. (2023). The gray mold spore detection of cucumber based on microscopic image and deep learning. Plant Phenomics 5, 11. doi: 10.34133/plantphenomics.0011 36930758 PMC10013786

[B12] Liao L. Zhou X. Li X. Yin Y. Chen K. Liu S. . (2026). Gynoecious and monoecious cucumbers drive the assembly of different rhizosphere microbial communities. Front. Plant Sci. 17, 1786995. doi: 10.3389/fpls.2026.1786995 41868524 PMC13002568

[B13] Liu J. Wang X. Chen Q. Yan P. Guo D. (2025). Intelligent deep learning architecture for precision vegetable disease detection advancing agricultural new quality productive forces. Front. Plant Sci. 16, 1611865. doi: 10.3389/fpls.2025.1611865 40880870 PMC12380761

[B14] Oh K. B. Chen Y. Matsuoka H. Yamamoto A. Kurata H. (1996). Morphological recognition of fungal spore germination by a computer-aided image analysis and its application to antifungal activity evaluation. J. Biotechnol. 45, 71–79. doi: 10.1016/0168-1656(95)00148-4

[B15] Pal M. Manimaran P. Panigrahi P. K. (2022). A multi scale time–frequency analysis on electroencephalogram signals. Physica A. Stat. Mechanics Its Appl. 586, 126516. doi: 10.1016/j.physa.2021.126516 38826717

[B16] Qian X. Zhang C. Chen L. Li K. (2022). Deep learning-based identification of maize leaf diseases is improved by an attention mechanism: Self-attention. Front. Plant Sci. 13, 864486. doi: 10.3389/fpls.2022.864486 35574079 PMC9096888

[B17] Qiao C. Li K. Zhu X. Jing J. Gao W. Zhang L. (2025). Detection of cucumber downy mildew spores based on improved YOLOv5s. Inf. Process. Agric. 12, 179–194. doi: 10.1016/j.inpa.2024.05.002 38826717

[B18] Runge F. Ndambi B. Thines M. (2012). Which morphological characteristics are most influenced by the host matrix in downy mildews? A case study in Pseudoperonospora cubensis. PloS One 7, e44863. doi: 10.1371/journal.pone.0044863 23166582 PMC3499517

[B19] Sasaki Y. Okamoto T. Imou K. Torii T. (1999). Automatic diagnosis of plant disease recognition between healthy and diseased leaf. J. Japanese Soc. Agric. Machinery 61, 119–126. doi: 10.1016/S1474-6670(17)42113-6

[B20] Seo Y. Park B. Yoon S. C. Lawrence K. C. Gamble G. R. (2018). Morphological image analysis for foodborne bacteria classification. Trans. ASABE 61, 5–13. doi: 10.13031/trans.11800

[B21] Shaw P. Uszkoreit J. Vaswani A. (2018). “ Self-attention with relative position representations”, in: Proceedings of the 2018 Conference of the North American Chapter of the Association for Computational Linguistics: Human Language Technologies, Volume 2 (Short Papers) (New Orleans, LA: Association for Computational Linguistics), 464–468. doi: 10.18653/v1/N18-2074

[B22] Sletova M. E. Korottseva I. B. Kameneva A. V. Engalycheva I. A. Belov S. N. (2024). Study of morphobiological characteristics of the pathogen causing true powdery mildew in the family Cucurbitaceae L. crops. Russ. Agric. Sci. 50, 694–701. doi: 10.3103/S1068367425700338

[B23] Uchida K. Takamatsu S. Matsuda S. So K. Sato Y. (2009). Morphological and molecular characterization of Oidium subgenus Reticuloidium (powdery mildew) newly occurred on cucumber in Japan. J. Gen. Plant Pathol. 75, 92–100. doi: 10.1007/s10327-009-0146-4 30311153

[B24] Vinogradova K. Dibrov A. Myers G. (2020). “ Towards interpretable semantic segmentation via gradient-weighted class activation mapping (student abstract)”, in: Proceedings of the AAAI Conference on Artificial Intelligence (Palo Alto, CA: AAAI Press), 34, 13943–13944. doi: 10.1609/aaai.v34i10.7244

[B25] Voita E. Talbot D. Moiseev F. Sennrich R. Titov I. (2019). “ Analyzing multi-head self-attention: Specialized heads do the heavy lifting, the rest can be pruned”, in: Proceedings of the 57th Annual Meeting of the Association for Computational Linguistics (Florence, Italy: Association for Computational Linguistics), 5797–5808. doi: 10.18653/v1/P19-1580

[B26] Wang X. Zhang S. Wang Z. Zhang Q. (2014). Recognition of cucumber diseases based on leaf image and environmental information. Trans. Chin. Soc. Agric. Eng. 30, 148–153. doi: 10.3969/j.issn.1002-6819.2014.14.019

[B27] Wu C. Wang X. (2017). Preliminary research on the identification system for anthracnose and powdery mildew of sandalwood leaf based on image processing. PloS One 12, e0181537. doi: 10.1371/journal.pone.0181537 28749977 PMC5531471

[B28] Young-Joon C. H. O. I. Seung-Beom H. O. N. G. Hyeon-Dong S. H. I. N. (2005). A re-consideration of Pseudoperonospora cubensis and P. humuli based on molecular and morphological data. Mycol. Res. 109, 841–848. doi: 10.1017/S0953756205002534 16121571

[B29] Zhang J. Li X. Tian J. Luo H. Yin S. (2023). An integrated multi-head dual sparse self-attention network for remaining useful life prediction. Reliability Eng. System Saf. 233, 109096. doi: 10.1016/j.ress.2023.109096 38826717

[B30] Zhang Q. L. Yang Y. B. (2021). “ Sa-net: Shuffle attention for deep convolutional neural networks”, in: ICASSP 2021-2021 IEEE International Conference on Acoustics, Speech and Signal Processing (ICASSP) (Piscataway, NJ, USA: IEEE), 2235–2239. doi: 10.1109/ICASSP39728.2021.9414568

[B31] Zhang D. Zhang W. Cheng T. Lei Y. Qiao H. Guo W. . (2024). Segmentation of wheat scab fungus spores based on CRF_ResUNet++. Comput. Electron. Agric. 216, 108547. doi: 10.1016/j.compag.2023.108547 38826717

[B32] Zhang Q. Zhou M. Wang J. (2022). Increasing the activities of protective enzymes is an important strategy to improve resistance in cucumber to powdery mildew disease and melon aphid under different infection/infestation patterns. Front. Plant Sci. 13, 950538. doi: 10.3389/fpls.2022.950538 36061767 PMC9428622

[B33] Zhao X. Li K. Li Y. Ma J. Zhang L. (2022). Identification method of vegetable diseases based on transfer learning and attention mechanism. Comput. Electron. Agric. 193, 106703. doi: 10.1016/j.compag.2022.106703 38826717

[B34] Zhao Y. Lin F. Liu S. Hu Z. Li H. Bai Y. (2019). Constrained-focal-loss based deep learning for segmentation of spores. IEEE Access 7, 165029–165038. doi: 10.1109/ACCESS.2019.2953085 25079929

